# Supplementary material for: The minimal meningococcal ProQ protein has an intrinsic capacity for structure-based global RNA recognition
Source: Nat Commun. 2020 Jun 4;11:2823. doi: 10.1038/s41467-020-16650-6 (PMC7272453; doi:10.1038/s41467-020-16650-6)
Supplement: Supplementary file 1 — Supplementary Information [file 41467_2020_16650_MOESM1_ESM.pdf]

Supplementary Information

The minimal meningococcal ProQ protein has an intrinsic capacity for structure-based global RNA recognition

S. Bauriedl et al.

SUPPLEMENTARY METHODS

Sequence retrieval. BlastN searches (http://www.ncbi.nlm.nih.gov/sutils/ genom_table.cgi) were used to get information for sequence alignments of the following genome sequences (accession numbers are given in parentheses): *N. meningitidis* 8013 (NC_017501), *N. meningitidis* MC58 (NC_003112), *N. meninigitidis* M22718 (NZ_CP016627), *N.meningitidis* α14 (NC_013016), *N. meninigitidis* Z2491 (NC_003116), *N. meninigitidis* 331401 (NZ_CP012694), *N.gonorrhoeae* MS11 (NC_022240), *N.lactamica* 020-06 (NC_014752).

Neisseria meningitidis **growth conditions.** All strains used (Supplementary Table 2) were either grown on Columbia blood agar plates (COS, bioMerieux) or on GC medium base (GCB) plates (Difco) containing Kellogg's supplement I and II (22.2 mM glucose, 0.68 mM glutamine, 0.45 mM co-carboxylase, 1.23 mM $Fe(NO_3)_3$; all from Sigma) and appropriate antibiotics (7 µg ml^{-1} erythromycin, 100 µg ml^{-1} kanamycin, 7 µg ml^{-1} chloramphenicol or 5 µg ml^{-1}). All solid cultures were incubated at 37 ˚C in a 5% CO_2 humidified atmosphere. For liquid cultures, *N.meningitidis* grown on plates over night, were harvested, and a starter culture was inoculated to a final optical density $OD_{600\,nm}$ of 1.0 in 5 ml GCB-liquid medium supplemented with Kellogg's supplement I and II and sodium bicarbonate (GCBL++) in 50 ml falcon-tubes. After one hour the starter culture was used to inoculate GCBL++ medium to a final $OD_{600\,nm}$ of 0.15. Bacteria were either grown in 50 ml or 200 ml Falcon-tubes at 37 °C without CO_2 for total RNA isolation.
Bacterial growth was measured fully automated using the Infinite 200 Pro plate reader (Tecan). The evening before the experiment, the meningococcal strains were streaked out on GCB plates supplemented with antibiotics if required and incubated for 18 h at 37 °C and 5% CO_2 in an incubator. Subsequently, 10 single colonies of each strain were collected with a swab and were dabed on fresh COS agar plates following incubation for 4 h at 37 °C and 5% CO_2. Afterwards, the bacteria were absorbed with swabs and used for inoculation of 3 ml $GCBL^{++}$ medium per strain. The $OD_{600\,nm}$ of each bacterial suspension was adjusted to an $OD_{600\,nm}$ of 0.1 by adding the required volume of GCBL++. Subsequently, each well of a 96-well microtiter plate was filled with 200 µl of the bacterial suspensions or $GCBL^{++}$ medium as a blank. For each strain under investigation, 4 wells of the 96-well microtiter plate were implemented with bacterial suspension. Growth in 96-well microtiter plates was monitored using the Infinite 200 Pro instrument (Tecan) at 37 °C with 3 mm amplitude shaking. Optical density was monitored (absorbance at 595 ± 10 nm) every 30 min for 12 h. For data analysis, a logistic growth model was fitted to the data by non-linear regression analysis using R version 2.7.0 and the nls package as described in [1]. Three biological replicates were performed of the experiment.

Construction of *Neisseria meningitidis* mutants. Supplementary Table 2 provides a list with all generated mutant strains by natural transformation of plasmids or PCR-amplified constructs carrying an antibiotic cassette. For natural transformation, a starter culture was inoculated with *N.meningitidis* cells grown overnight on solid media until an $OD_{600\ nm}$ of 1.0 was reached. After incubation for one hour at 37 °C and 200 rpm, the starter culture was diluted in 1ml GCBL++ media to an $OD_{600\ nm}$ of 0.15 in 10 ml Falcon-tubes and mixed with 500 ng to 1,000 ng plasmid or purified PCR product. After growing the culture to late logarithmic growth phase at 37 °C and 200 rpm, the bacterial cells were pelleted and plated on GCB agar plates with appropriate antibiotics. Mutants were checked by colony-PCR, Southern blot and sequencing.

Construction of *N. meningitidis* Δ*proQ*, Δ*hfq* and Δ*proQ*Δ*hfq* *and* *proQ*::*3xFLAG* strains by plasmids. Supplementary Table2 provides a list with all generated mutant strains, Supplementary Table 3 lists all plasmids and Supplementary Table 4 all oligonucleotides used in this study. The *proQ* gene (NMV_0698) was deleted from 8013 strain by replacing the coding sequence by the insertion of the chloramphenicol resistance cassette *catGC* as described in [2]. First, p8013_ΔproQ::cat plasmid was constructed in *E.coli* Top10 cells. Upstream and downstream flanking regions of the *proQ* gene were amplified by PCR from chromosomal DNA using primer pairs 1241/1242 and 1243/1244, respectively. The resulting upstream flanking region was digested with *BamH*I and *Hind*III while the flanking downstream region was digested with *Hind*III and *Xho*I. The *catGC* cassette was generated by digestion of pTnMax5 with *Hind*III. The predigested upstream and downstream flanking regions and the *catGC* cassette were cloned into the *Bam*HI and *Xho*I predigested pBluescript II SK (+) vector to yield the p8013_ΔproQ::cat plasmid. The plasmid was checked by colony PCR with primer pair kb9/329. The plasmid was used for natural transformation in *N. meningitidis* 8013 strain. Positive chloramphenicol-resistant clones were checked by PCR on gDNA using JVO-13303/JVO-13305, resulting in strain Δ*proQ*.

The *hfq* gene (NMV_1689) was deleted from strain 8013 by replacing the coding sequence by the insertion of the kanamycin resistance cassette *aphA-1*. First, p8013_Δhfq::aphA-1 plasmid was constructed in Top10 cells. Upstream and downstream flanking regions of the *hfq* gene were amplified by PCR from chromosomal DNA using primer pairs 1237/1238 and 1239/1240, respectively. The resulting upstream flanking region was digested with *BamH*I and *EcoR*I while the flanking downstream region was digested with *EcoR*I and *HindII*I. The *aphA-1* cassette was generated

by digestion of pUC4K with *EcoR*I. The predigested upstream and downstream flanking regions and *aphA-1* cassette were cloned into the *Bam*HI and *Hind*III predigested pBluescript II SK (+) vector to yield plasmid p8013_Δhfq::aphA1. The plasmid was checked by colony PCR with primer pair kb9/329. The plasmid was used for natural transformation in *N. meningitidis* 8013 and the Δ*proQ* strain to obtain the double knockout strain Δ*proQ*Δ*hfq* . Positive kanamycin-resistant clones were checked by PCR on gDNA using JVO-11603/JVO-11502, resulting in strain Δ*hfq and* Δ*proQ*Δ*hfq*.

To construct a *proQ::3xFLAG*-tagged strain, a plasmid *(3xFLAG::aphA-1*) containing the 3xFLAG and the *aphA-1* cassette flanked by 500 nt up- and downstream of the *proQ* stop codon was cloned into *E. coli* as described in [2]. First, 500 nt upstream of the *proQ* stop codon were amplified by PCR from chromosomal DNA of 8013 with primers JVO-13234 and JVO-13235 containing the 3xFLAG sequence at its 3´end. The resulting PCR product was digested with *BamH*I and *EcoR*I. Next, the downstream region of the *proQ* gene was amplified by PCR from chromosomal DNA of 8013 with primers JVO-13236 and JVO-13237 followed by digestion with *EcoR*I and *Hind*III. The plasmid pUC4K was then digested with *EcoR*I to obtain the *aphA-1* kanamycin cassette. Both fragments and the *aphA-1* cassette were cloned into the vector pBluescript II SK (+) predigested with *Bam*HI and *Hind*III. The generated pproQ-3xFLAG::aphA-1 plasmid was checked by colony PCR with primer pair kb9/329. This plasmid was then used for transformation of strain 8013. Transformants were verified by colony PCR on gDNA using JVO-11502/JVO-11603 and in-frame fusion of *proQ*::*3xFLAG* by sequencing with JVO-13238, respectively.

Construction of *N. meningitidis* complementation strains by overlap PCR. All *N. meningitidis* complementation strains are listed in Supplementary Table 2 and were constructed by overlap PCR as described in [3] and [2]. For primer details see Supplementary Table 4 including the strategy for the combinatory set-up to generate the PCR fragmens. The strains were generated by transformation of the obtained overlap PCR fragments into the *lctP* and *aspC* locus of strain 8013. All PCR products carried fragments encoding the target *gene::3xFLAG* including its native promotor and tzhe erythromycin resistance cassettes. These PCR products were flanked by ~500 bp of homologous sequences of the *in trans* complementation locus situated in the intergenic region between NMV_1884 and NMV_1885. As an example, for the complementation of Δ*proQ*, the *proQ* complementation construct was generated by overlap PCR as follows: A PCR fragment of 500 bp upstream of the intergenic region using upstream region primers (JVO-14059/1395) and a second PCR fragment of ~400 bp downstream of the intergenic region using downstream region primers

(1396/JVO-12664) were amplified from chromosomal DNA of strain 8013. A PCR fragment of *proQ::3xFLAG* including its native promotor was amplified using the target gene primers JVO-14061/JVO-14129 from chromosomal DNA of *proQ::3xFLAG*. The resistance cassette was amplified from plasmid pgcc2 using primers JVO-14130/JVO-14153 (EryR). The 5' ends of the antisense upstream region primer 1395, the antisense target gene primer JVO-14129 and sense downstream region primer 1396 contained ~25 bp of sequence homologous to the sense or antisense primer of the nearby fragment. All four PCR products were purified and then combined in a ratio of 50:50:50:50 ng to a 50 µl Phusion polymerase (Fermentas)-containing PCR reaction with sense and antisense primers (JVO-14059/JVO-12664) at a final concentration of 0.06 µM. Overlap PCR was performed using the following cycling conditions: 1 cycle of [98 °C, 1 min; 61 °C, 1 min; 72 °C, 10 min; 98 °C, 1 min], 40 cycles of [98 °C, 15 s; 57 °C, 30 s; 72 °C, 1 min], followed by a 10 min final extension at 72 °C. Knockout strains were transformed with purified PCR fragment and transformants were isolated by selction on erythromycin. All complementation strains were verified by PCR using JVO-14067/JVO-12664 which bind outside of the region used for cloning. Complementation strains for Δ*proQ* and Δ*proQ*Δ*hfq* were constructed similarly (see Supplementary Data 5).

Purification of ProQ protein. ProQ was cloned into the pTYB11 expression plasmid using *Spe*I and *Not*I restriction sites to allow the intein-based expression and purification of a tagless protein. Expression was done in *E.coli* BL21(DE3) in LB media and cells were induced with 0.5 mM IPTG at an $OD_{600\ nm}$ of 0.5 and cultivated overnight at 15 °C. All following purification steps were performed at 4 °C. The cells were lysed in 10 ml per 1 g pellet buffer A (20 mM HEPES pH 8.0, 500 mM NaCl, 1 mM EDTA, 0,1% Tween20, 10% Glycerol) by sonification with addition of DNase and Protease Inhibitor and the soluble fraction was obtained by ultra centrifiguation. After the calibration of the Chitin Beads (NEB Biolabs) with 10 column volume (CV) buffer B (20 mM HEPES pH 8.0, 500 mM NaCl, 1 mM EDTA, 10% Glycerol) the soluble fraction was loaded to the column two times. Washing was performed 3 times with 4 CV buffer C (20 mM HEPES pH 8.0, 500 mM NaCl, 1 mM EDTA, 10% Glycerol, 5 mM ATP, 5 mM $MgCl_2$ and 0.1 mg/mL inactivated *E.coli* Lysate) and a 10 min incubation. For eluation the column was first equilibrated with 25 CV buffer D (20 mM HEPES pH 8.0, 500 mM NaCl, 1 mM EDTA, 10% Glycerol, 50 mM DTT) and then incubated 40 h at 4 °C with ~2 CV buffer D.

All eluted fractions with an absorption 260nm/280nm ratio under 2 were combined, concentrated and purified by size exclusion chromatography (HiLoad 16/600 Superdex 75 pg column, GE Healthcare) in buffer E (20 mM HEPES pH 8.0, 250 mM NaCl, and 2 mM DTT).

PNPase purification *pnp* was amplified from *N. meningitis* genomic DNA with primers JVO-16889/JVO-16890) and cloned into pET-M14(+) (*kanR*, amplification primers JVO-16887/JVO-16888) backbone plasmid with an N-terminal His-tag and 3C-cleavage site by site and ligation independent cloning through T4-DNA-Polymerase. *E.coli* BL21-RIL cells were transformed with pETM14_H6-3C-NmPNPase (pMiG_008) and grown in LB media with kanamycin and chloramphenicol to an OD_{600nm} of 0.6 at 37 °C. Expression of PNPase was induced by 0.5 mM IPTG and the cells were grown overnight at 18 °C. Cells were pelleted and frozen as a pellet at -80 °C. The pellet was resuspended in 40 ml lysis buffer (50 mM sodium phosphate pH 8.0, 1 M NaCl, 0.1 mM EDTA, 2 mM 2-ME, 20 mM imidazole, 1 mM PMSF) and sonicated for cell lysis. The lysate was cleared at 15,000 × *g* for 20 min at 4 °C. The supernatant was used as sample and 500 µl Protino Ni-IDA beads (Macherey-Nagel) were added and incubated at over-head rotation at 4 °C for 30 min. Beads were recovered by centrifugation at 500 × *g*, and washed three times with 30 ml lysis buffer. H6-3C-PNPase was eluted with 2 CV elution buffer (lysis buffer +280 mM imidazole, pH 8.0). The eluate was rebuffered and concentrated by centrifugal filtration (30 K cut-off, Amicon, Merck-Millipore) in storage buffer (20 mM HEPES/NaOH pH 7.4, 100 mM NaCl, 2 mM 2-ME, 20% (w/v) glycerol), and flash frozen in liquid nitrogen and stored at -80 °C. Protein concentration was determined by absorption utilizing the extinction coefficient 35,995 $mM^{-1} cm^{-1}$.

RNA degradation assay DNA templates containing a T7 promoter sequence were generated by PCR and served as templates for *in vitro* transcription. Oligos used to generate the individual DNA templates are listed in Supplementary Table 4. T7 transcription was carried out with the MEGAscript® T7 kit (Ambion) according to the manufactures´ protocol. Degradation assays were conducted in reaction buffer (20 mM Tris/HCl, pH 7.5, 100 mM NaCl, 1 mM $MgCl_2$, 1 mM DTT). Degradation assays were performed at 37°C using 0.2 µM PNPase, 0.2 µM RNA and 1 µM purified ProQ protein per reaction. *In vitro* transcribed RNA was heated for 2 min at 50 °C and slowly cooled down to room temperature. ProQ and preheated RNA were preincubated together at room temperature before adding PNPase. To stop the reactions, equal volumes of RNA loading dye were added and after denaturation at 95 °C for 5 min whole samples were resolved on a 10% polyacrylamide gel containing 7 M urea. Gels were stained with ethidiumbromide.

Cell culture and cell adhesion experiments The human nasopharyngeal epithelial cell line Detroit562 (ATCC® number CCL-138™) was used for the cell adhesion assays. Cells were grown in Eagle's minimum essential medium (EMEM) (Lonza) with 10% feta calf serum (Thermo Fisher), 1% nonessential amino acids (Lonza) and 1% sodium pyruvate (Lonza) (EMM+++) at 37 °C and 5% CO_2. Cell adhesion assays were performed as described in ref. 2. Briefly, Detroit562 cells were seeded on 24-well tissue culture plates (Corning Costar) at a density of 5×10^4 cells and grown to $\approx1\times10^5$ prior to infection. Cells were infected for 4 h with bacteria grown to midlogarithmic growth phase ($OD_{600\ nm}$: 0.5) in EMM+++ media at a multiplicity of infection (MOI) of 20. Four wells per strain were used to determine the "invasive" or "gentamicin protected" bacteria and treated with 0.2 mg ml^{-1} gentamicin (Biochrom, Berlin, Germany) for 1 h at 37 °C to kill extracellulat bacteria. Another 4 wells per strain were used to assess the "adherent" or "cell associated" bacteria and treated with 1% saponin (Serva, Heidelberg, Germany) for 15 min to break up the Detroit562 cells. The number of adherent bacteria in the supernatant [N(adhesion)] was determined as colony-forming units (CFU) by plating serial dilutions of the lysates of blood agar. For each strain, the adhesion rate (N(adhesion) $N(total)^{-1}$) was calculated as the number of colony forming units (CFU) recovered after 4 h of infection divided by the seeded CFU [N(total)] defined in parallel. One hour post gentamicin treatment the "invasive" bacteria were determined the same way. For each strain, the invasion rate [N(invasion) $N(adhesion)^{-1}$] was calculated as the number of colony forming units (CFU) recovered after gentamicin treatment [N(invasion)] divided by the number of adherent bacteria [N(adhesion)] defined in parallel. Experiments were repeated at least three times.

Static biofilm assay The static biofilm assay protocol was modified from [4]. The strains were streaked on COS agar and incubated at 37 °C with 5% CO_2 for not longer than 12 h to avoid biofilm formation on the plate. The bacterial cells were suspended GCBL++ medium to an $OD_{600\ nm}$ of 0.1. For each strain, 100 µl of the bacterial suspension were added to 8 wells of a 96-well microtiter plate. Additional 8 wells were each filled with 100 µl GCBL++. The microtiter plate was incubated at 37 °C with 5% CO_2 for 17.5 h. Afterwards, the medium was removed from all wells prior to adding 100 µl 0.05% crystal violet and incubating the plate for 10 min at room temperature to stain the biofilm. After removing the dye, each well was washed twice with 200 µl PBS. Next 100 µl 100% ethanol were added per well prior to incubation for 20 min at room temperature. After mixing by pipetting, the $OD_{600\ nm}$ was measured with the ELISA reader Multiskan® EX (Thermo Scientific). The values of 7 wells, which showed the lowest variance, were used for calculation. The cut-off was determined as three times the average of the $OD_{600\ nm}$ of the wells filled with medium only. The experiment was repeated twice.

Enzyme-linked immunosorbent assay (ELISA) The ELISA was performed as described in ref. [5] using the monoclonal anticapsule antibody Mab 24 [5] which specifically detects the *N. meninigitidis* serogroup C capsule polysaccharide. For the ELISA, meningococci were grown overnight on solid media and resuspended in 1×PBS to a final OD_{600nm} of 0.15. Of each bacterial suspension, 20 µl were added to each well of a microtiter plate (Greiner) which had been coated with 25 ng ml^{-1} Poly-D-lysine (Sigma) before. The plates were dried at room temperature prior to fixing the bacteria with 100 µl/well PBS–0.05% glutaraldehyde for 10 min at room temperature. After blocking nonspecific binding sites by incubation with PBS–1% bovine serum albumin (BSA) for 1 h at 37 °C (150 µl/well), binding of the anticapsule antibody was performed for 1 h in 1% BSA/PBS (20 µl/well; antibody dilution 1:4,000 in 1% BSA/PBS). After three wash steps with PBS, incubation with the secondary peroxidase-conjugated anti-mouse immunoglobulin antibody (Dianova) was performed for 1 h in 1% BSA/PBS (20 µl/well; antibody dilution 1:2,500 in 1% BSA/PBS) at room temperature. Photometric measurement was performed at OD_{414nm} with the ELISA reader Multiskan® EX (Thermo Scientific).

Serum bactericidal assay. Serum bactericidal assay were performed three times with the *N. meninigitidis* strains as described in [6]. *N. meninigitidis* strains grown overnight on solid media were resuspended in Veronal-buffered saline (VBS) (5 mM 5,5-diethyl-barbituric acid [Serva, Heidelberg, Germany], 145 mM NaCl, 0.5 mM $MgCl_2$, 0.15 mM $CaCl_2$) plus 0.5% bovine serum albumin (BSA) (Sigma) (VSA). A total amount of 10^5 CFU was resuspended in VSA and either 0% Normal human serum (NHS) or 40% NHS in a final reaction voulume of 350 µl followed by incubation at 37 °C for 60 min. The reaction was stopped by incubation on ice and serial dilutions of the samples were plated on blood agar plates and incubated overnight at 37 °C with 5% CO_2. The experiment was repeated twice. Bacterial counts are given as log_{10}CFU per milliliter. Normal human serum was collected from healthy individuals without meningococcal vaccination. Fresh blood samples were centrifuged for 5 min at 2,000 × g and the obtained serum was snap-frozen in liquid nitrogen and stored at –80 °C.

SUPPLEMENTARY FIGURES

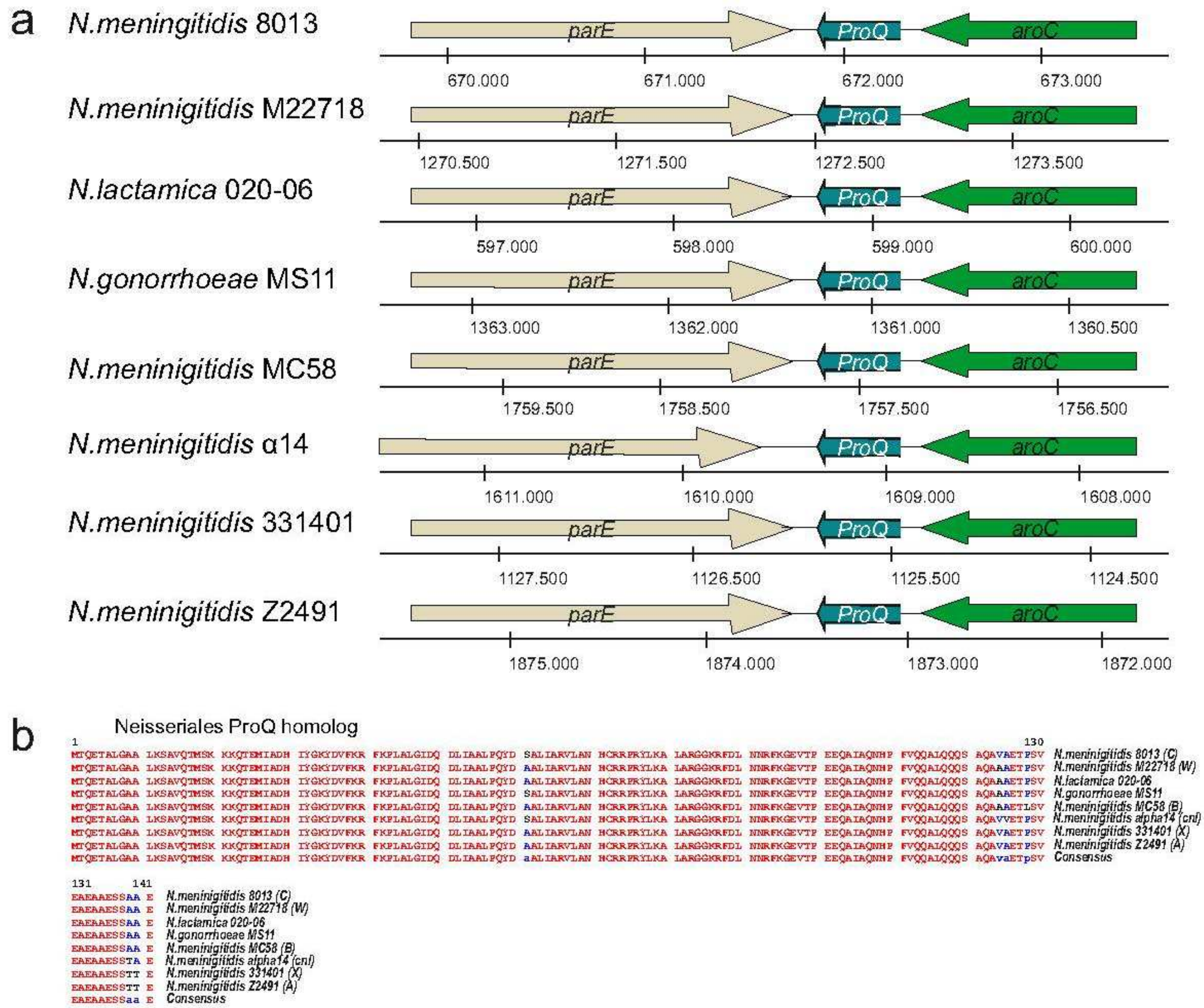

Supplementary Figure 1: Genomic location of meningococcal ProQ. a, Schematic illustration of the ProQ locus in the *N. meninigitidis* strains 8013, MC58, Z2491, α14, M22718 and 331401 representing distinct meningococcal serogroups, *N. lactamica* strain 020-06 and *N. gonorrhoeae* strain MS11. The nucleotide numbers below indicate genomic positions in each genome. **b**, Amino acid sequence alignment of ProQ from *N. meninigitidis* strains 8013, MC58, Z2491, α14, M22718 and 331401, reflecting distinct meningococcal serogroups given in parentheses, *N. lactamica* strain 020-06 and *N. gonorrhoeae* strain MS11. The degree of conservation is indicated by the colour of the

nucleotides (red: high conservation; blue: little conservation, black: little or no conservation). The alignment was created using MultAlin (http://multalin.toulouse.inra .fr/multalin/multalin.html).

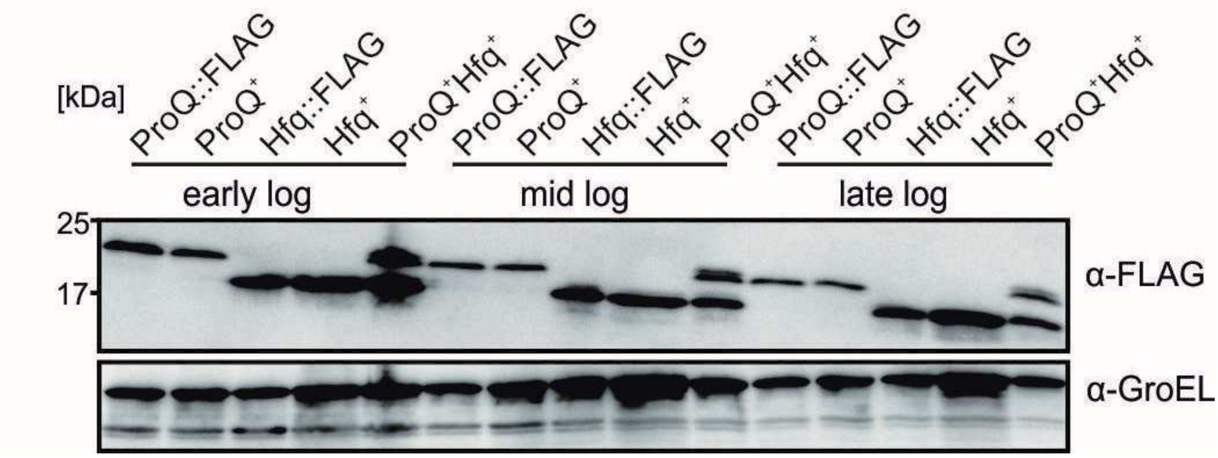

Supplementary Figure 2: Expression analysis of Hfq and ProQ in complemented Δ*proQ*, Δ*hfq* and Δ*proQ*Δ*hfq* strains. Equal amounts of cells ($OD_{600\ nm}$ = 0.01) of chromosomally FLAG-tagged *proQ* (ProQ::FLAG), *hfq* (Hfq::FLAG) and complemented deletion strains for *proQ* ($ProQ^+$) , *hfq* (Hfq^+) and *proQhfq* ($ProQ^+$ Hfq^+) were analyzed in three growth phases (early log: $OD_{600\ nm}$ 0.5, mid log: $OD_{600\ nm}$ 1.5, late log: $OD_{600\ nm}$ 2.0) by Western blotting with mouse anti-FLAG antibodies. Hfq and ProQ were complemented with *hfq* or *proQ* genes provided with their native promoters together with a C-terminal FLAG-tag into the *lctP* and *aspC* locus of *N. meninigitidis* strain 8013, respectively.

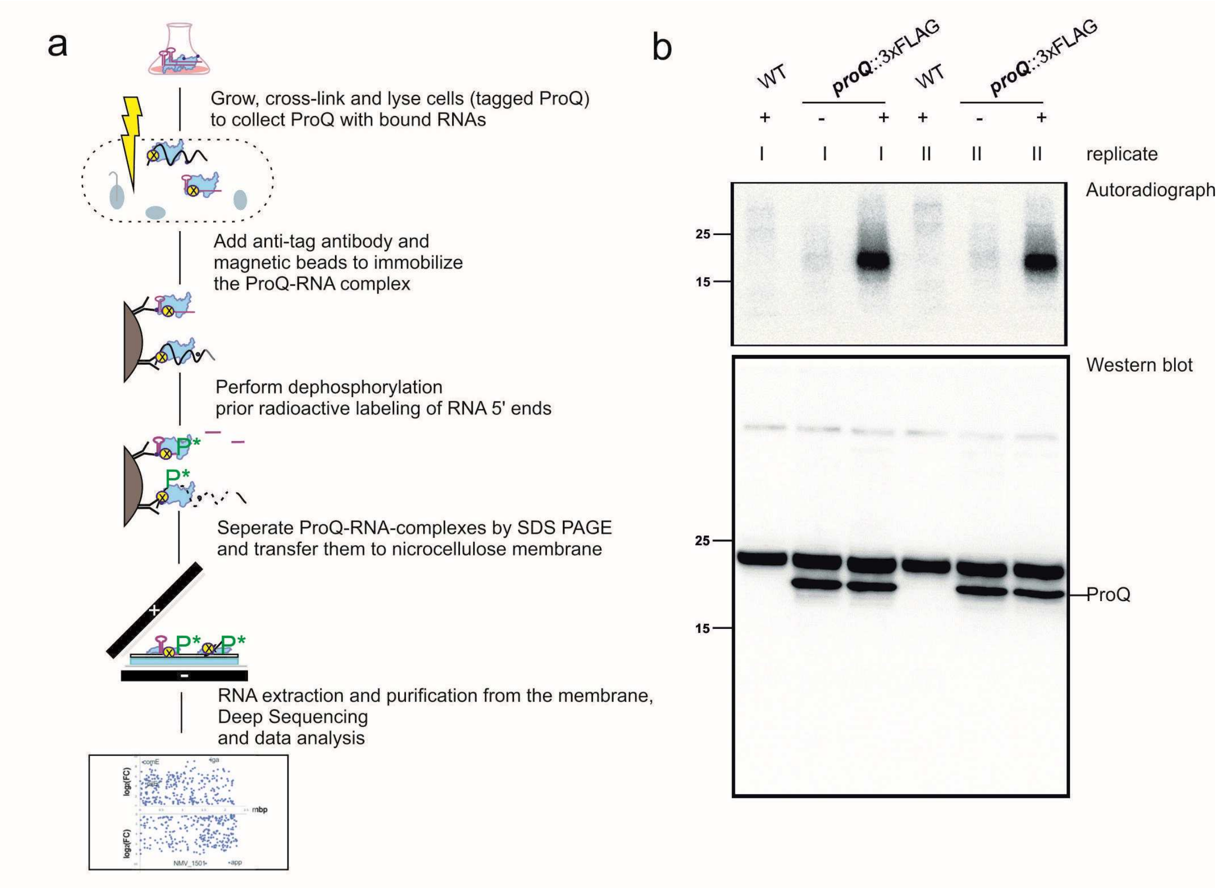

Supplementary Figure 3: UV CLIP-seq of the RNA-binding protein ProQ in *N. meningitidis* strain 8013. a, Schematic workflow of the UV CLIP-seq protocol performed with *N. meningitidis* expressing 3×FLAG tagged ProQ grown in GCBL++ media to late logarithmic growth phase ($OD_{600\ nm}$: 2.0). **b**, (Top panel) Autoradiograph of radioactively labeled RNA–protein complexes after transfer to nitrocellulose membranes. (Bottom panel) Detection of ProQ-3×FLAG proteins by Western blot using a mouse anti-FLAG antibody served as a control for successful immunoprecipitation. Shown are the obtained signals for the crosslinked wild-type (wt) and non-crosslinked 3×FLAG tagged *proQ* strain serving as negative controls and the cross-linked 3×FLAG tagged ProQ expressing strain in two independent experiments. Crosslinked samples are indicated by (+), non-cross-linked samples by (-) and the two biological replicates by (I, II).

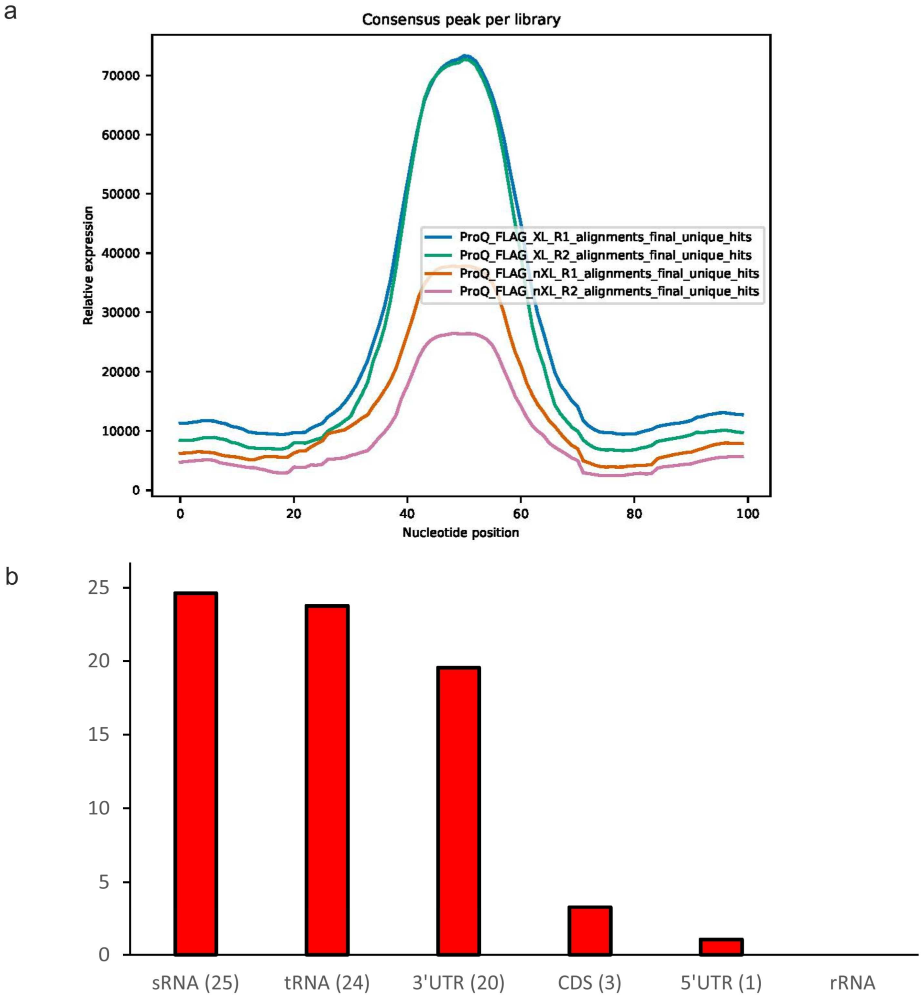

Supplementary Figure 4: Statistics on UV CLIP-seq of the RNA-binding protein ProQ in *N. meningitidis* strain 8013. a, Consensus peak per library of the obtained ProQ UV CLIP-seq data from two independent experiments. **b**, Histogram depicting the fraction of annotated RNA features with a ProQ CLIP-peak in percent among all features in the indicated RNA feature class.

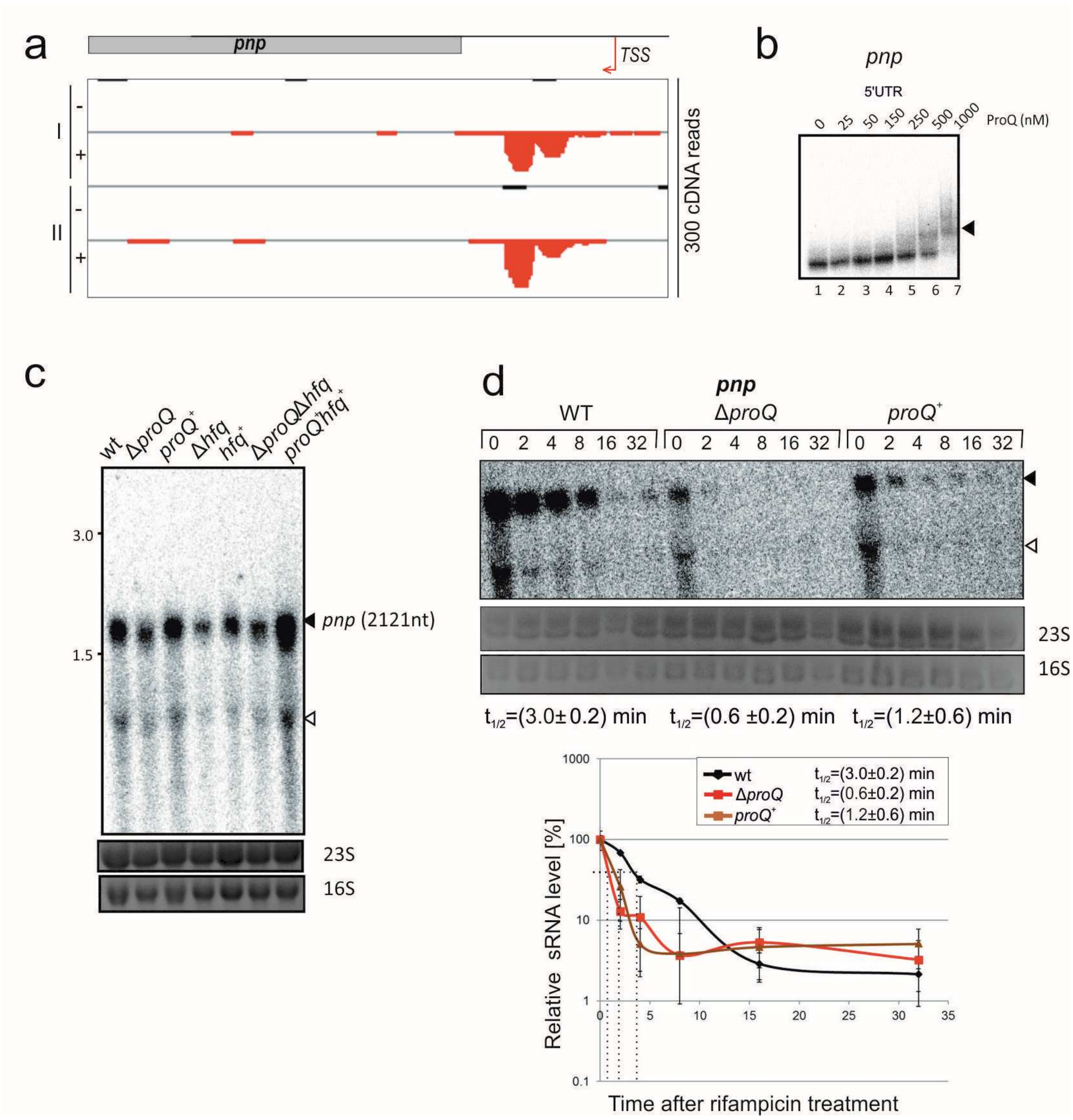

Supplementary Figure 5: Verification of ProQ-associated mRNA *pnp*. a, Detection of association of ProQ with *pnp* derived from UV CLIP-seq read coverages mapped to the 5'UTR. Cross-linked samples are indicated by (+), non-cross-linked samples by (-) and the two biological replicates by (I, II). **b**, *In vitro* gel-shift assays of ProQ and *pnp*. Migration of 0.04 pmol *in vitro* transcribed and ^{32}P-labeled RNA in a non-denaturating gel after incubation for 20 min with varying concentrations of purified ProQ protein (lane 1–7: 0, 25, 50, 150, 250, 500, 1,000 nM). Arrows indicate the RNA-protein complex. **c,** Expression analysis of candidate ProQ-associated mRNA *pnp* in *N. meningitidis* strain 8013. Total RNA was extracted at late logarithmic ($OD_{600\ nm}$ of 2.0) growth phase from wild-type (WT), Δ*proQ,* complemented *proQ*+, Δ*hfq*, complemented *hfq*+, Δ*proQ*Δ*hfq* and complemented *proQ*+*hfq*+ strains and analyzed by northern blot using labeled DNA probes complementary to the

mRNA (see Supplementary Table 9). Filled triangles highlight mRNA bands derived from TSS and open triangles highlight bands derived from processing. The housekeeping 16S and 23S rRNA served as loading control. **d**, RNA half-lives for *pnp* determined in *N. meningitidis* 8013 wild-type (WT), Δ*proQ* and complemented *proQ*$^{+}$ strains. Northern blots of total RNA extracted at the indicated time points (in minutes) after addition of rifampicin (250 µg/ml) are shown. 16S and 23S rRNA was used as loading control. The experiment was performed in triplicate, and the estimated half-life ($t_{1/2}$) of the respective sRNA in each strain is given at the bottom of each gel image plus/minus (±) one standard deviation. For the determination of mRNA half-lives, the relative sRNA level obtained by northern blotting and quantified with the ImageQuant software is shown on the y-axis, and the time after rifampicin treatment on the x-axis. Error bars represent the standard deviation calculated from at least two biological replicates. The insert depicts the estimated half-lifes ($t_{1/2}$) for the respective sRNA in each strain plus/minus (±) one standard deviation.

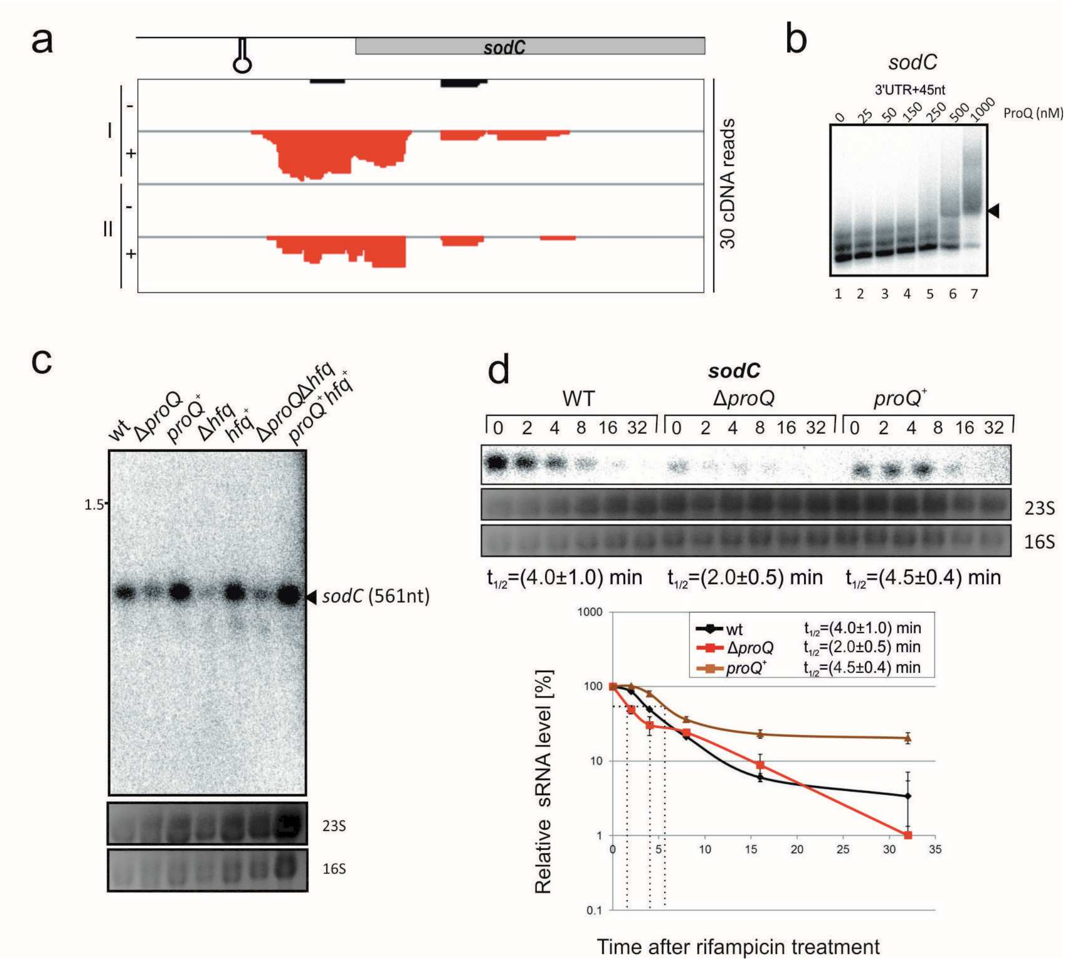

Supplementary Figure 6: Verification of ProQ-associated mRNA *sodC*. **a**, Detection of association of ProQ with *sodC* derived from CLIP-seq read coverages mapped to the CDS and 3'UTR. Cross-linked samples are indicated by (+), non-cross-linked samples by (-) and the two biological replicates by (I, II). **b**, *In vitro* gel-shift assays of ProQ and *sodC*. Migration of 0.04 pmol *in vitro* transcribed and ^{32}P-labeled RNA in a non-denaturating gel after incubation for 20 min with varying concentrations of purified ProQ protein (lane 1–7: 0, 25, 50, 150, 250, 500, 1,000 nM). Arrows indicate the RNA-protein complex. **c,** Expression analysis of candidate ProQ-associated mRNA *sodC* in *N. meningitidis* strain 8013. Total RNA was extracted at late logarithmic ($OD_{600\ nm}$ of 2.0) growth phase from wt, Δ*proQ,* complemented *proQ*$^+$, Δ*hfq*, complemented *hfq*$^+$, Δ*proQ*Δ*hfq* and complemented *proQ*$^+$*hfq*$^+$ strains and analyzed by northern blot using labeled DNA probes complementary to the mRNA (see Supplementary Table 9). Filled triangles highlight mRNA bands derived from TSS and open triangles highlight bands derived from processing. The housekeeping 16S and 23S rRNA served as loading control. Quantification of relative mRNA expression levels obtained from three independent biological replicates are indicated. Error bars represent the standard deviation calculated from three

biological replicates. **d**, RNA half-lives for *sodC* determined in *N. meningitidis* 8013 wt, Δ*proQ* and complemented *proQ*$^{+}$ strains. Northern blots of total RNA extracted at the indicated time points (in minutes) after addition of rifampicin (250 µg ml^{-1}) are shown. 16S and 23S rRNA was used as loading control. The experiment was performed in triplicate, and the estimated half-life ($t_{1/2}$) of the respective sRNA in each strain is given at the bottom of each gel image plus/minus (±) one standard deviation. For the determination of mRNA half-lives, the relative sRNA level obtained by northern blotting and quantified with the ImageQuant software is shown on the y-axis, and the time after rifampicin treatment on the x-axis. Error bars represent the standard deviation calculated from at least two biological replicates. The insert depicts the estimated half-lifes ($t_{1/2}$) for the respective sRNA in each strain plus/minus (±) one standard deviation.

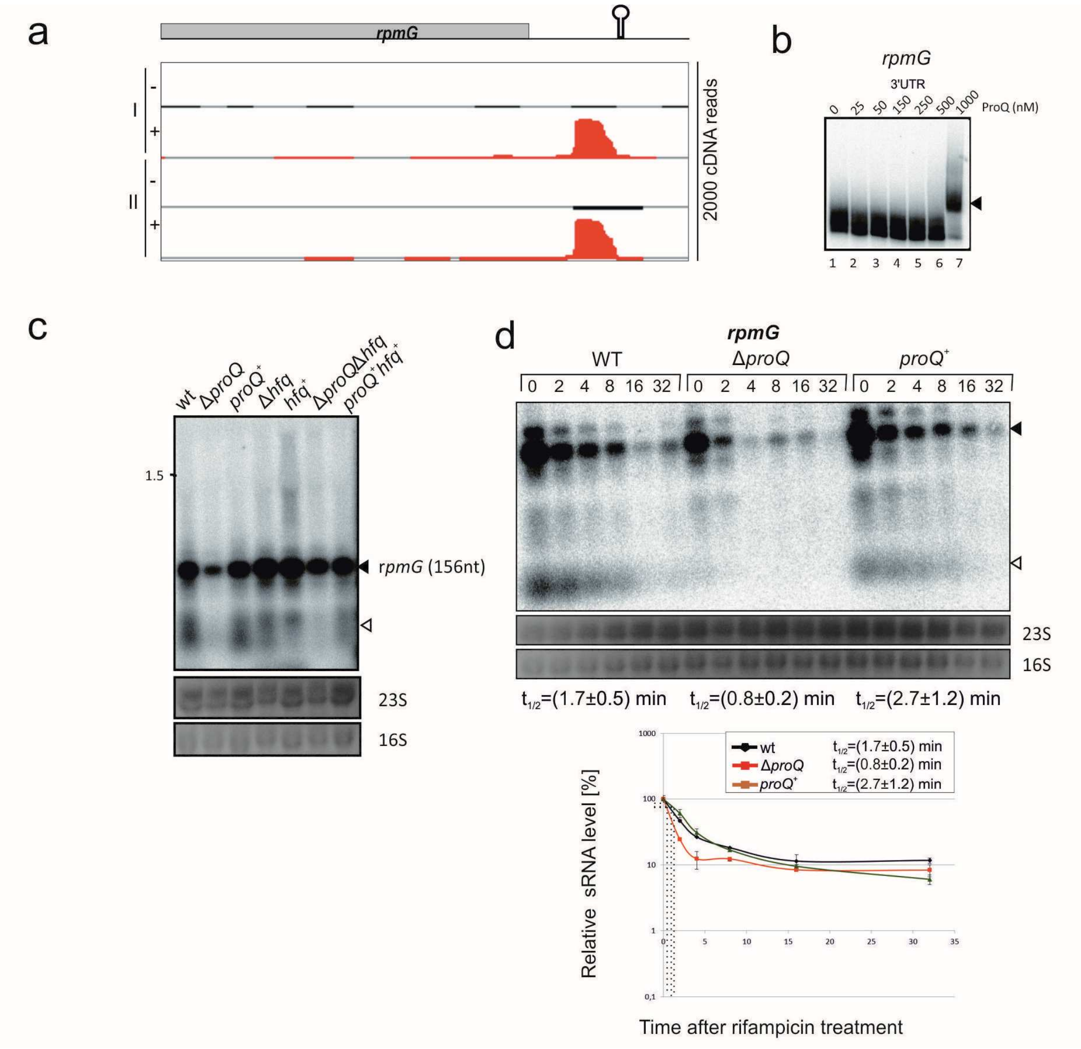

Supplementary Figure 7: Verification of ProQ-associated mRNA *rpmG*. a, Detection of association of ProQ with *rpmG* derived from UV CLIP-seq read coverages mapped to the 3'UTR. Cross-linked samples are indicated by (+), non-cross-linked samples by (-) and the two biological replicates by (I, II). **b**, *In vitro* gel-shift assays of ProQ and *rpmG*. Migration of 0.04 pmol *in vitro* transcribed and ^{32}P-labeled RNA in a non-denaturating gel after incubation for 20 min with varying concentrations of purified ProQ protein (lane 1–7: 0, 25, 50, 150, 250, 500, 1,000 nM). Arrows indicate the RNA-protein complex. **c**, Expression analysis of candidate ProQ-associated mRNA *rpmG* in *N. meningitidis* strain 8013. Total RNA was extracted at late logarithmic ($OD_{600\,nm}$ of 2) growth phase from wild-type (wt), Δ*proQ,* complemented *proQ*$^+$, Δ*hfq*, complemented *hfq*$^+$, Δ*proQ*Δ*hfq* and complemented *proQ*$^+$*hfq*$^+$ strains and analyzed by northern blot using labeled DNA probes complementary to the mRNA (see Supplementary Table 9). Filled triangles highlight mRNAs bands derived from TSS and open triangles highlight bands derived from processing. The housekeeping 16S and 23S rRNA served as loading

control. Quantification of relative mRNA expression levels obtained from three independent biological replicates are indicated. Error bars represent the standard deviation calculated from three biological replicates. (bottom panel) **d**, RNA half-lives for *rpmG* determined in *N. meningitidis* 8013 wt, Δ*proQ* and complemented *proQ*$^{+}$ strains. Northern blots of total RNA extracted at the indicated time points (in minutes) after addition of rifampicin (250 µg ml^{-1}) are shown. 16S and 23S rRNA was used as loading control. The experiment was performed in triplicate, and the estimated half-life ($t_{1/2}$) of the respective sRNA in each strain is given at the bottom of each gel image plus/minus (±) one standard deviation. For the determination of mRNA half-lives, the relative sRNA level obtained by northern blotting and quantified with the ImageQuant software is shown on the y-axis, and the time after rifampicin treatment on the x-axis. Error bars represent the standard deviation calculated from at least two biological replicates. The insert depicts the estimated half-lifes ($t_{1/2}$) for the respective sRNA in each strain plus/minus (±) one standard deviation.

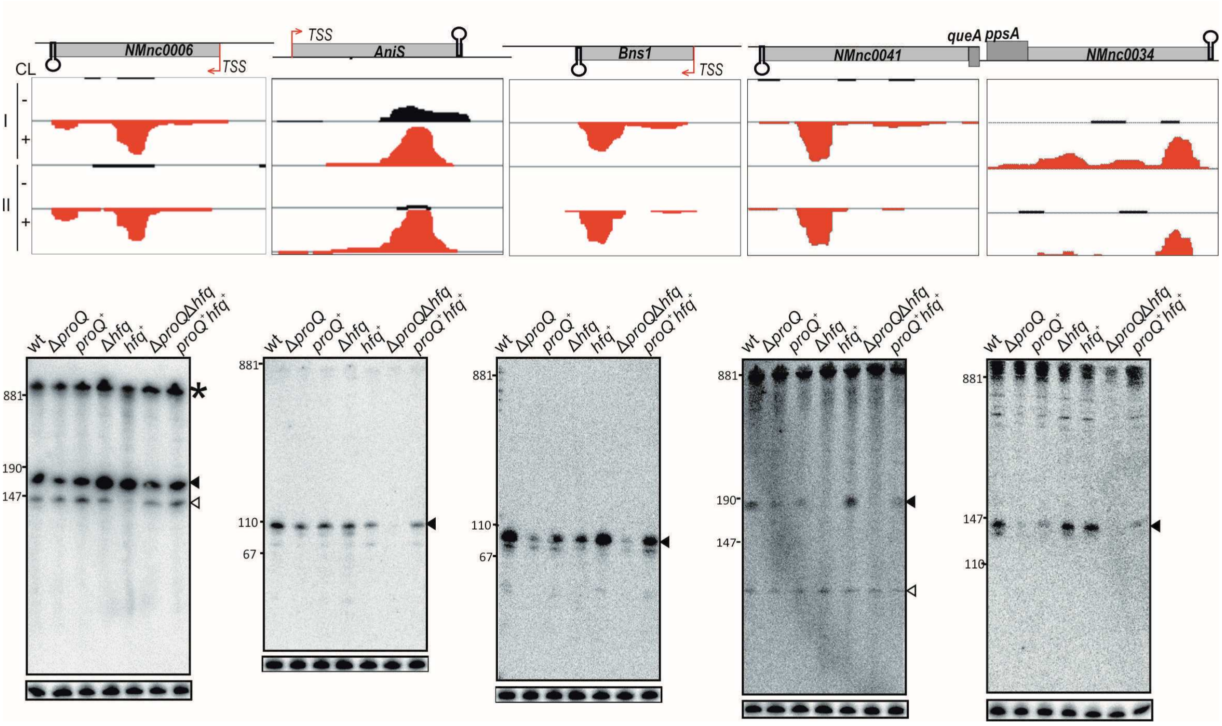

Supplementary Figure 8: Verification of ProQ-associated sRNAs. (top panel) Detection of ProQ association with sRNAs (NMnc0006, AniS, Bns1, NMnc0041, NMnc0034) derived from UV CLIP-seq read coverages mapped to genomic sRNA sequences. Cross-linked samples are indicated by (+), non-cross-linked samples by (-) and the two biological replicates by (I, II). (bottom panel) Expression analysis of candidate ProQ-associated sRNAs in *N. meningitidis* 8013 by northern blot analysis. Total RNA was extracted at late logarithmic (OD_{600} of 2.0) growth phases from wild-type (wt), Δ*proQ,* complemented *proQ*+, Δ*hfq*, complemented *hfq*+, Δ*proQ*Δ*hfq* and complemented *proQ*+*hfq*+ strains and analyzed by northern blot using labeled DNA probes complementary to the sRNAs (see Supplementary Table 9). Filled triangles highlight sRNAs bands derived from TSS, open triangles highlight bands derived from processing and asterisks highlight signals of unclear origin. The housekeeping 5S rRNA served as loading control at the bottom.

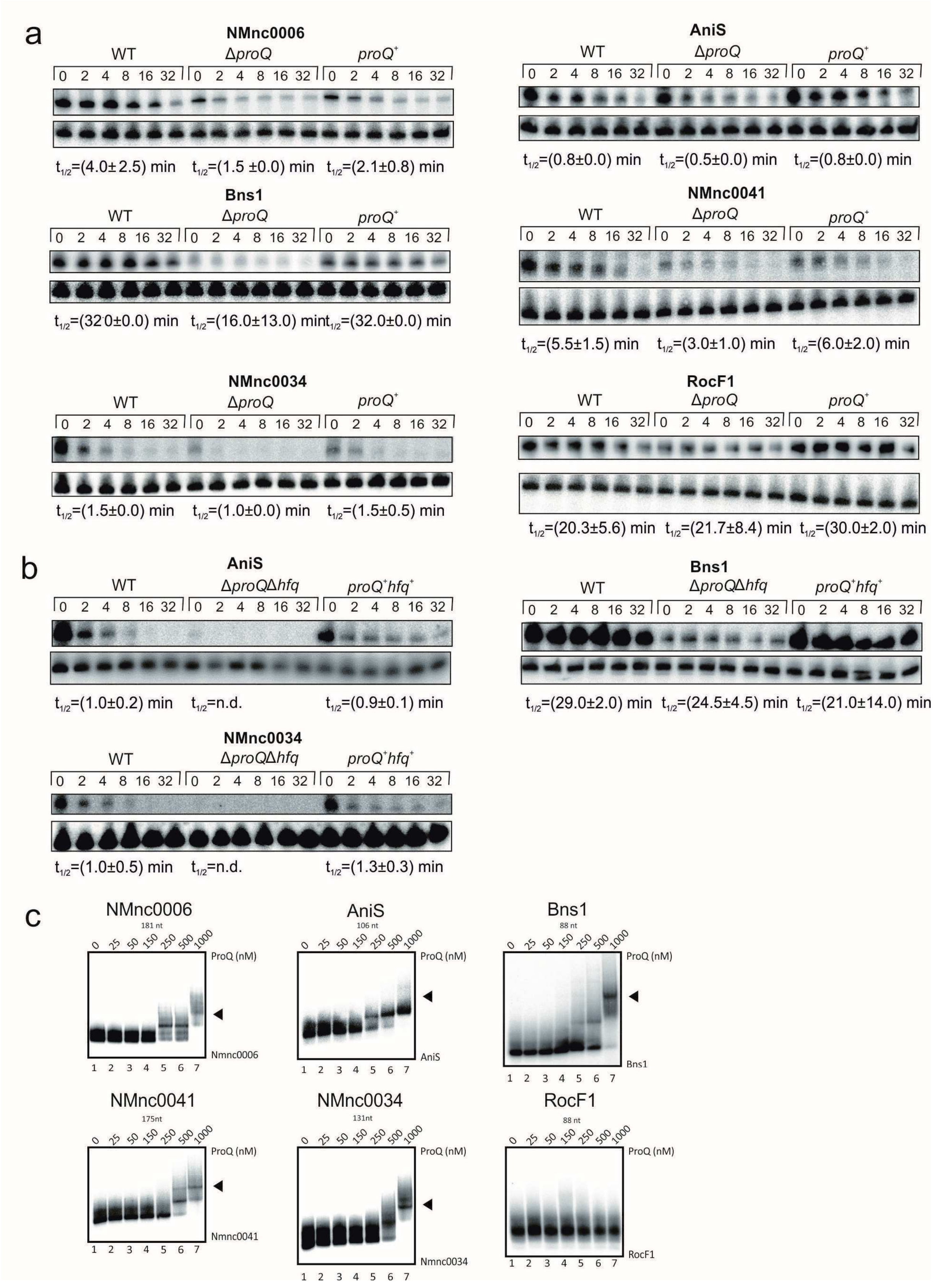
a
NMnc0006
WT ΔproQ proQ+
0 2 4 8 16 32 0 2 4 8 16 32 0 2 4 8 16 32
t1/2=(4.0± 2.5) min t1/2=(1.5 ±0.0) min t1/2=(2.1±0.8) min
AniS
WT ΔproQ proQ+
0 2 4 8 16 32 0 2 4 8 16 32 0 2 4 8 16 32
t1/2=(0.8±0.0) min t1/2=(0.5±0.0) min t1/2=(0.8±0.0) min
Bns1
WT ΔproQ proQ+
0 2 4 8 16 32 0 2 4 8 16 32 0 2 4 8 16 32
t1/2=(32 0±0.0) min t1/2=(16.0±13.0) mint1/2=(32.0±0.0) min
NMnc0041
WT ΔproQ proQ+
0 2 4 8 16 32 0 2 4 8 16 32 0 2 4 8 16 32
t1/2=(5.5±1.5) min t1/2=(3.0±1.0) min t1/2=(6.0±2.0) min
NMnc0034
WT ΔproQ proQ+
0 2 4 8 16 32 0 2 4 8 16 32 0 2 4 8 16 32
t1/2=(1.5±0.0) min t1/2=(1.0±0.0) min t1/2=(1.5±0.5) min
RocF1
WT ΔproQ proQ+
0 2 4 8 16 32 0 2 4 8 16 32 0 2 4 8 16 32
t1/2=(20.3±5.6) min t1/2=(21.7±8.4) min t1/2=(30.0±2.0) min
b
AniS
WT ΔproQΔhfq proQ+hfq+
0 2 4 8 16 32 0 2 4 8 16 32 0 2 4 8 16 32
t1/2=(1.0±0.2) min t1/2=n.d. t1/2=(0.9±0.1) min
Bns1
WT ΔproQΔhfq proQ+hfq+
0 2 4 8 16 32 0 2 4 8 16 32 0 2 4 8 16 32
t1/2=(29.0±2.0) min t1/2=(24.5±4.5) min t1/2=(21.0±14.0) min
NMnc0034
WT ΔproQΔhfq proQ+hfq+
0 2 4 8 16 32 0 2 4 8 16 32 0 2 4 8 16 32
t1/2=(1.0±0.5) min t1/2=n.d. t1/2=(1.3±0.3) min
c
NMnc0006
181 nt
0 25 50 150 250 500 1000
ProQ (nM)
Nmnc0006
1 2 3 4 5 6 7
AniS
106 nt
0 25 50 150 250 500 1000
ProQ (nM)
AniS
1 2 3 4 5 6 7
Bns1
88 nt
0 25 50 150 250 500 1000
ProQ (nM)
Bns1
1 2 3 4 5 6 7
NMnc0041
175nt
0 25 50 150 250 500 1000
ProQ (nM)
Nmnc0041
1 2 3 4 5 6 7
NMnc0034
131nt
0 25 50 150 250 500 1000
ProQ (nM)
Nmnc0034
1 2 3 4 5 6 7
RocF1
88 nt
0 25 50 150 250 500 1000
ProQ (nM)
RocF1
1 2 3 4 5 6 7

Supplementary Figure 9: ProQ stabilizes associated sRNAs. **a**, sRNA half-lives for AniS, Bns1, NMnc006 NMnc0041 and NMnc0034 determined in *N. meningitidis* 8013 wild-type (WT), Δ*proQ* and complemented *proQ*$^+$ strains. Northern blots of total RNA extracted at the indicated time points (in minutes) after addition of rifampicin (250 μg ml^{-1}) are shown. For each strain, each lane corresponds to one time point as indicated on top of the respective lane. 5S rRNA was used as loading control. The sRNA RocF1 which is associated with Hfq but not ProQ served as a negative control. Each experiment was performed in duplicate or triplicate and quantifications of gel images obtained for sRNA half-lives are summarized in Supplementary Figure 10. The estimated half-life ($t_{1/2}$) of the respective sRNA in each strain is given at the bottom of each panel plus/minus (±) one standard deviation. **b**, sRNA half-lives for AniS, Bns1 and NMnc0034 determined in *N. meningitidis* 8013 wild-type (WT), Δ*proQ*Δ*hfq* and complemented *proQ*$^+$*hfq*$^+$ strains. Northern blots of total RNA extracted at the indicated time points (in minutes) after addition of rifampicin (250 μg ml^{-1}) are shown. 5S rRNA was used as loading control. Each experiment was performed in duplicate or triplicate and quantifications of gel images obtained for sRNA half-lives are summarized in Supplementary Figure 10. The estimated half-life ($t_{1/2}$) of the respective sRNA in each strain is given at the bottom of each panel plus/minus (±) one standard deviation. **c**, *In vitro* gel-shift assays of ProQ and ProQ-associated sRNAs (AniS, Bns1, NMnc006, NMnc0041 and NMnc0034). Migration of 0.04 pmol *in vitro* transcribed and ^{32}P-labeled RNA in a non-denaturating gel after incubation for 20 min with varying concentrations of purified ProQ protein (lane 1–7: 0, 25, 50, 150, 250, 500, 1000 nM). Arrows indicate the RNA-protein complex. *In vitro* gel-shift assay with ProQ-independent sRNA RocF1 and increasing concentrations of purified ProQ protein as a negative control.

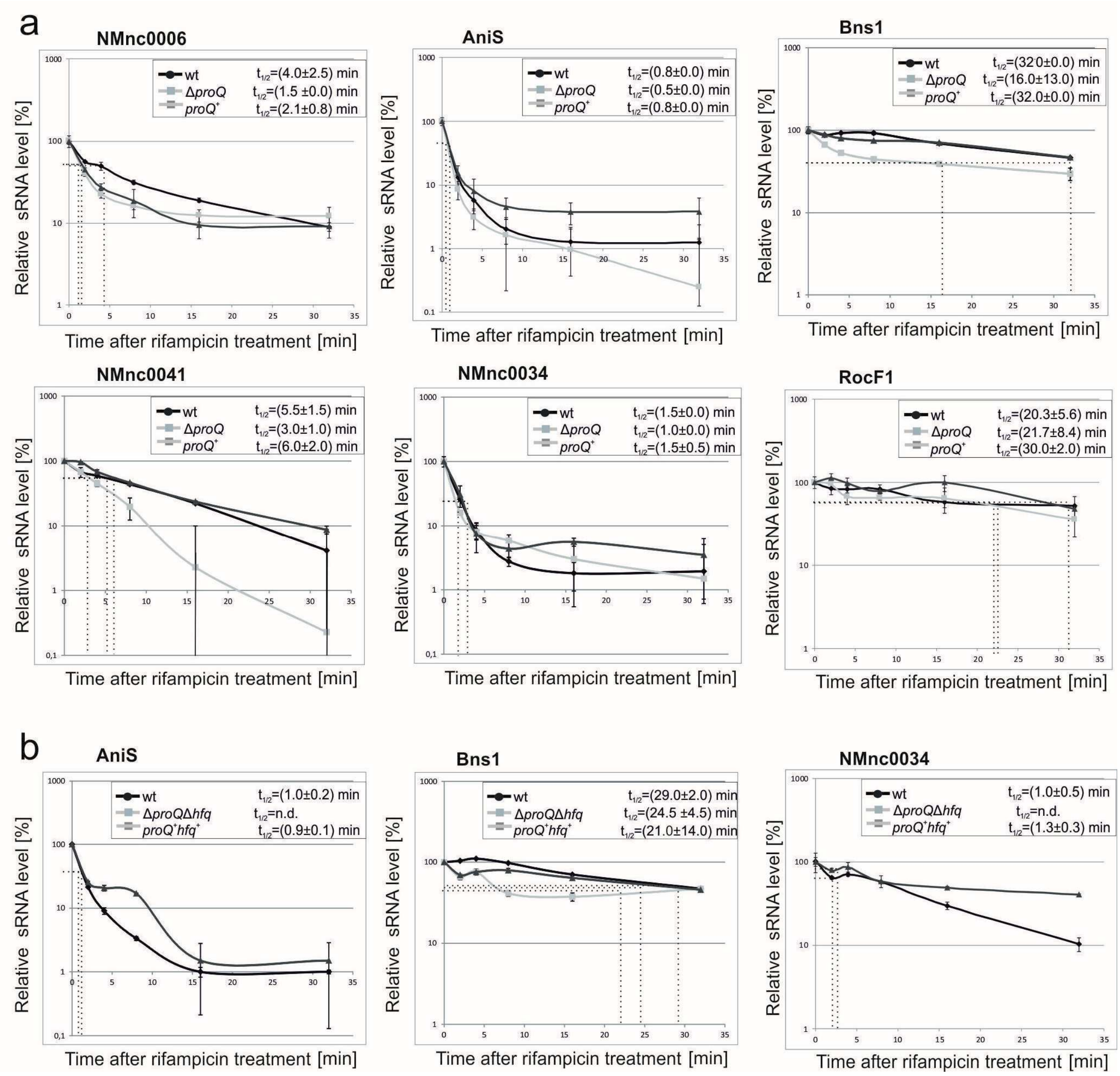

Supplementary Figure 10: Quantifications of stability of ProQ binding sRNAs. a, sRNA half-lives were determined for NMnc006, AniS, Bns1, NMnc0041 and NMnc0034. in *N. meningitidis* 8013 wild-type (wt), Δ*proQ* and complemented *proQ*+ strains in rifampicin RNA stability assays. **b**, sRNA half-lives were determined for AniS, Bns1 and NMnc0034. in *N. meningitidis* 8013 wt, Δ*proQ*Δ*hfq* and complemented *proQ*+*hfq*+ strains. In each panel, the relative sRNA level determined by northern blotting and quantified with the ImageQuant software is shown on the y-axis, and the time after rifampicin treatment on the x-axis. Error bars represent the standard deviation calculated from at least two biological replicates. In each panel, the insert depicts the estimated half-lifes ($t_{1/2}$) for the respective sRNA in each strain plus/minus (±) one standard deviation.

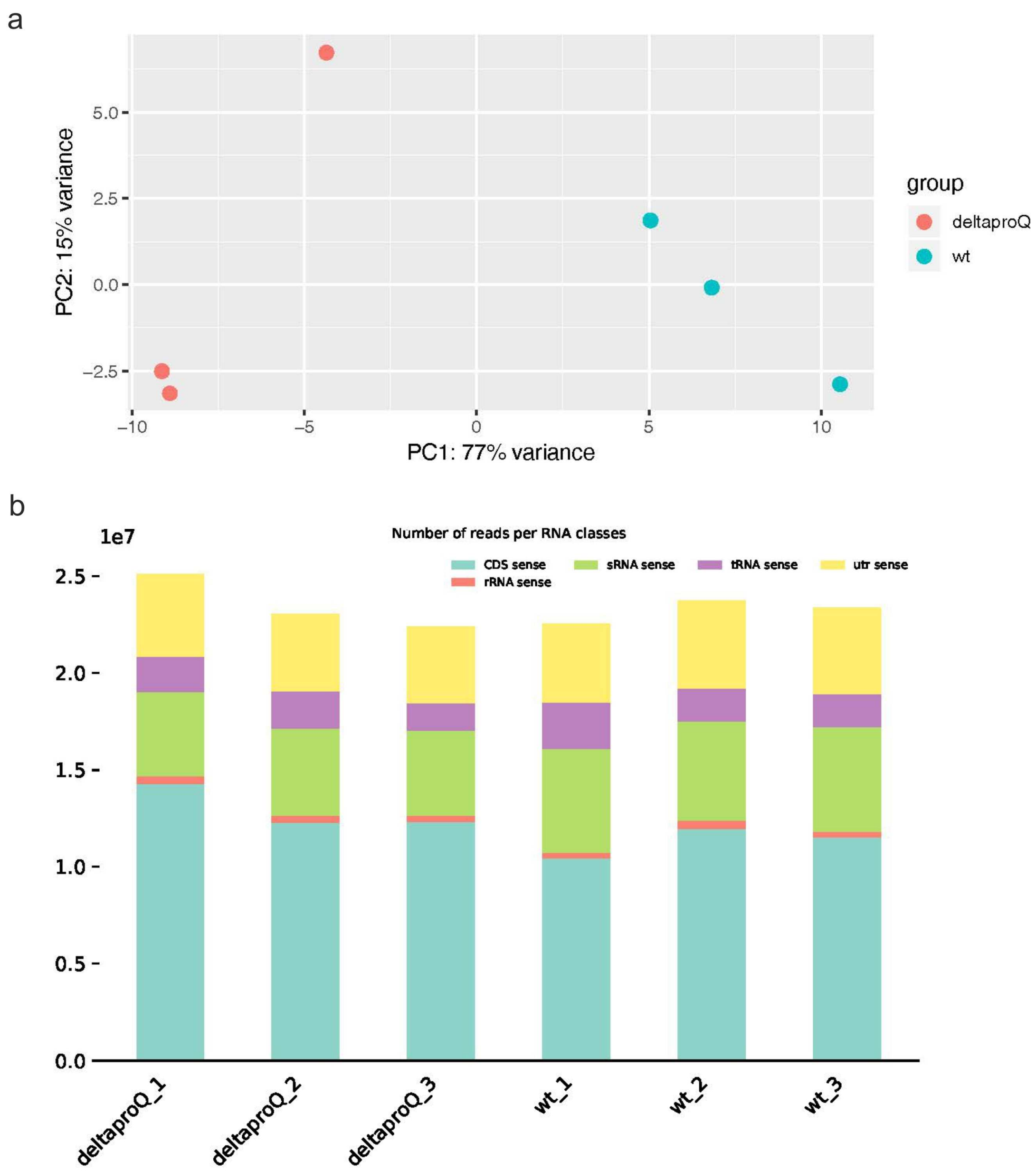

Supplementary Figure 11: Sequencing statistics of transcripts identified as regulated in the RNA‐seq data of the Δ*proQ* mutant compared to the wild-type strain *N.meninigitidis* 8013. a, The principle component analysis carried out on the three replicates of Δ*proQ* ("deltaproQ", red dots) and wild-type ("wt", light blue dots) samples, showing principle components (PC) 1 and 2 on the x- and y-axis, respectively, which together account for more than 90% of the observed gen expression variation between both strains. **b**, Stacked bar chart representing the percent of aligned reads on the

y-axis classified by RNA categories for the three replicates of Δ*proQ* ("deltaproQ") and WT ("wt") samples as depicted on the *x*-axis.

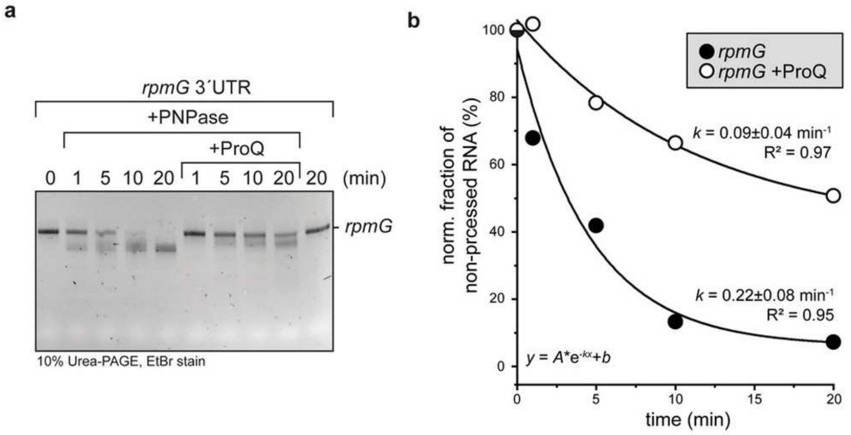

Supplementary Figure 12: ProQ protects targeted *rpmG* RNA from PNPase degradation. (a) *rpmG* 3'UTR was protected by ProQ from PNPase degradation (direct ProQ target, Figure 2a, Figure 6a). n = 2. RNA samples were analysed on a 10% polyacrylamide gel containing 6 M urea and stained with ethidiumbromid (EtBr). (b) ProQ decreased the PNPase-dependent degradation rate by ~2-fold for the direct target *rpmG* 3´UTR. Band intensities in (a) were densitometrically quantified and the normalized fraction of non-processed RNA (y-axis, in % of the total RNA per lane) plotted agains time (x-axis, in min). The degradation kinetics could be fitted by single exponential decay function ($y = A * e^{-k*x} + b$, k is the rate constant, A and b are constants)

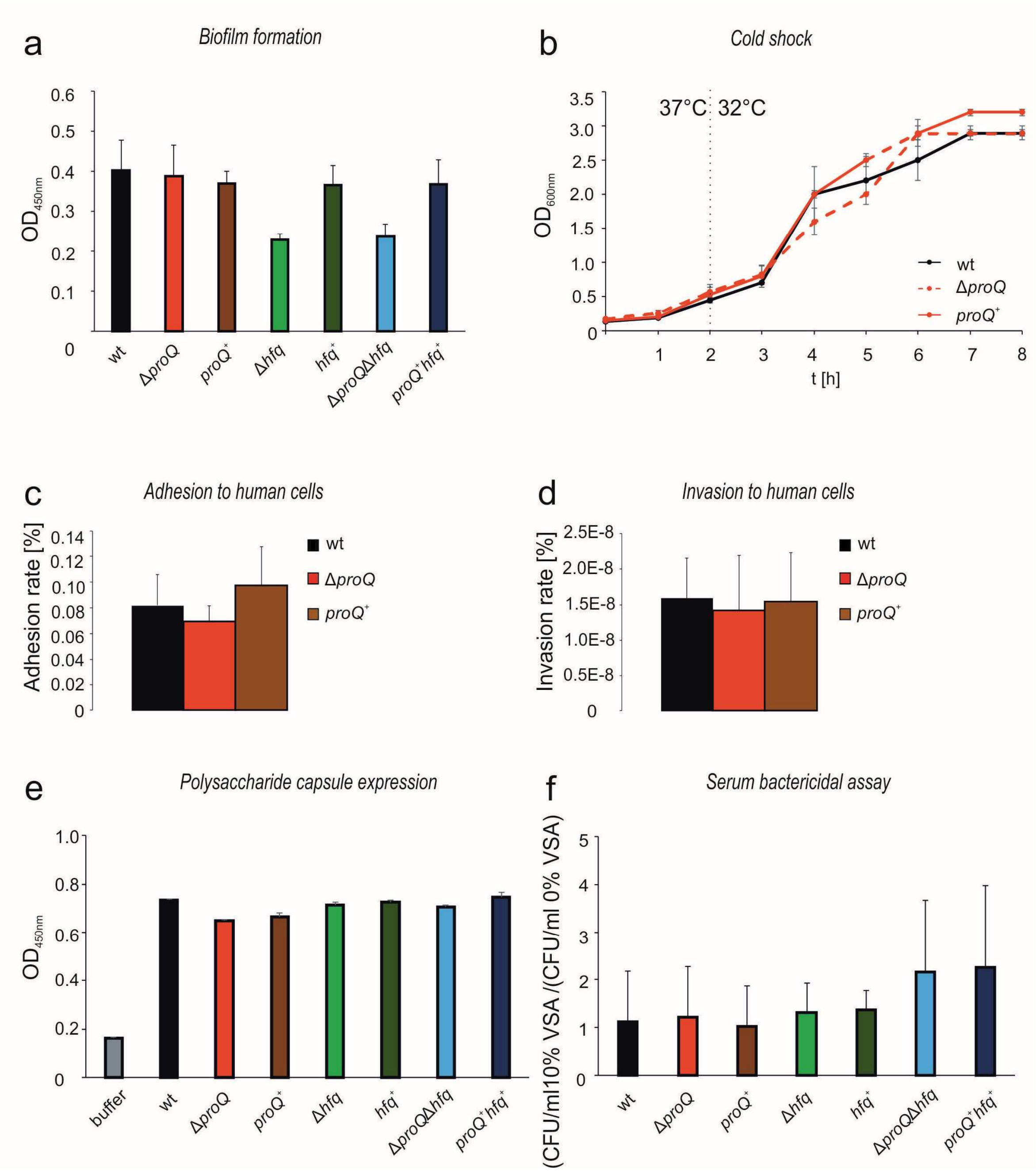

Supplementary Figure 13: **ProQ is not required for adhesion to human epithelial cells and serum resistance**. **a**, Static biofilm formation of 8013 wild-type, mutant and corresponding complemented strains. The diagram shows the measured $OD_{450\ nm}$ values of the stained biofilm for each strain minus the blank. Error bars indicate the standard deviation from two independent experiments. **b**, Growth of *N. meningitidis* 8013 wild-type, Δ*proQ* and complemented *proQ*+ strains at 32 °C in GCBL++. Growth as quantified by change in OD_{600} is given on the x-axis and the time *t* in hours

on the y-axis. The arrow indicates the time point of changing temperature from 37 °C to 32 °C. Error bars indicate the standard deviation from three independent experiments. **c**, Adhesion and **d**, invasion rates of 8013 wild-type, Δ*proQ* and complemented *proQ*$^{+}$ strains in the human nasopharyngeal epithelial cell line Detroit562. Shown is the average adhesion (panel **c**) and invasion rate (paneld **d**) of each mutant strain given on the y- axis from three independent experiments. The error bars represent the standard error of the mean. **e**, Serogroup C polysaccharide capsule expression of *N. meninigitidis* strains 8013 wild-type (wt), mutant and corresponding complemented mutant strains determined by enzyme-linked immunosorbent assay (ELISA). Shown are bar diagrams representing the mean of three independent experiments. The error bars represent the standard error of the mean. **f**, Serum bactericidal assay of *N. meninigitidis* strains 8013 wild-type, mutant and corresponding complemented strains. For each strain the average number of colony forming units (CFU) in VSA buffer with 10% normal human serum (NHS) relative to the number of CFU in VSA buffer without NHS (0%) is given on the y- axis as determined from three independent experiments. The error bars represent the standard error of the mean. For experimental details see SUPPLEMENTAL METHODS.

Abbreviations: wt, wild-type strain; Δ*proQ*, *proQ* deletion strain; *proQ*$^{+}$, *proQ* deletion strain complemented *in trans*; Δ*hfq*, *hfq* deletion strain; *hfq*$^{+}$, *hfq* deletion strain complemented *in trans*; Δ*proQ*Δ*hfq*, *proQ* and *hfq* double deletion strain; *proQ*$^{+}$*hfq*$^{+}$, proQ and hfq deletion strain complemented *in trans*.

SUPPLEMENTARY TABLES

Supplementary Table 1: Nucleotide sequence conservation of *proQ* in *N. meningitidis*

The analysis comprised nucleotide sequences of *N. meninigitidis* strains 8013, MC58, Z2491, α14, M22718 and 331401 for each housekeeping gene used for multi locus sequence typing (*abcz*, *adk*, *aroE*, *fumC*, *gdh*, *pdhC*, *pgm*) and *proQ*. Nucleotide diversity π was calculated using Tajima's Neutrality Test [7]. Codon positions included were 1st+2nd+3rd+Noncoding. All ambiguous positions were removed for each sequence pair (pairwise deletion option). Sequence analyses were conducted in MEGA X [8]. The nucleotide diversity of *proQ* in the six strains is even below the average nucleotide diversity for the other six housekeeping genes (0.035265), demonstrating the high conservation of *proQ*.

**Gene**	**Nucleotide diversity π**
abcz	0.046523
adk	0.011490
aroE	0.039095
fumC	0.031198
gdh	0.034901
pdhC	0.016116
pgm	0.067534
proQ	0.026604

Supplementary Table 2: Bacterial strains used in this study.

Strain names	Relevant genotypes	Resistance	Source
N. meningitidis	All *N. meningitidis* strains have 8013 background		
8013	Wild type	-	IHM strain collection
Δ*proQ*	*proQ*::*cat,* Deletion of *proq* (NMV_0689)	Cm^R	This study
proQ$^+$	*proQ*::*cat,* lctP::proQ-3xFLAG::erm::aspCD, complementation of *proq* (NMV_0689)	Erm^R Cm^R	This study
Δ*hfq*	*hfq*::*cat,* Deletion of *hfq* (NMV_1646)	Cm^R	(Heidrich et al. 2017)
Δ*hfq*	*hfq*::*aphA-3,* Deletion of *hfq* (NMV_1646)	Km^R	This study
hfq$^+$	*hfq*::, lctP::hfq-3xFLAG::erm::aspCD, complementation of *hfq* (NMV_1646)	Erm^R Km^R	This study
Δ*proQ*Δ*hfq*	*proQ*::*cat,* Deletion of *proq* (NMV_0689); *hfq::aphA-3, Deletion of hfq (NMV_1646)*	Cm^R Km^R	This study
proQ$^+$*hfq*$^+$	*proQ*::*cat* , *hfq aphA-3* C, lctP::proQ-3xFLAG hfq-3xFLAG::erm::aspCD, complementation of *proq* (NMV_0689) and *hfq* (NMV_1646)	Erm^R Cm^R Km^R	This study
ProQ-3xFLAG	*proQ*-3xFLAG::*aphA-1* C-terminal 3xFLAG tag at native locus (NMV_0689) in 8013 background	Km^R	This study
Hfq-3xFLAG	*hfq*-3xFLAG::*aphA-1* C-terminal 3xFLAG tag at native locus (NMV_1646) in 8013 background	Km^R	(Heidrich et al. 2017)
ProQ-3xFLAG/ Δ*hfq*	*proQ*-3xFLAG::*aphA-1* C-terminal 3xFLAG tag, *hfq*::*cat*	Km^R Cm^R	This study
Hfq-3xFLAG/ Δ*proQ*	*hfq*-3xFLAG::*aphA-1* C-terminal 3xFLAG tag, *proQ*::*cat*	Km^R Cm^R	This study
E. coli strains			
TOP10	*mcrA Δ(mrr-hsdRMS-mcrBC) Φ80lacZ ΔM15 ΔlacX74 deoR recA1 araD139 Δ(ara-leu)7697 galU galK rpsL endA1 nupG pgaA*	Str^R	Invitrogen

Str^R: streptomycin resistant; Kan^R: kanamycin resistant; Cm^R: chloramphenicol resistant; Erm^R: erythromycin resistant

Supplementary Table 3: Plasmids used in this study

**Plasmid trivial name**	**comment**	**Resistance**	**Reference**
pGCC2	pGCC2 empty vector harbouring an erythromycin resistance cassette	Erm^R Km^R	[9]
pGG1	Plasmid (based on pZE12-luc) harbouring a non-polar *aphA-3* kanamycin resistance cassette	Km^R AmpR	[3]
pUC4K	Plasmid carrying an *aphA-1* kanamycin resistance cassette	Km^R	GE healthcare
pBluescript II SK(+)	Cloning vector	Amp^R	Invitrogene
pTnMax5	Plasmid harbouring a catGC chloramphenicol resistance cassette	Km^R,Cm^R	
p8013_Δ*proQ*::*cat*	Plasmid (based on pBluescript II SK(+)) harbouring construct for generating knockout of proQ	Cm^R	This study
p8013_Δ*hfq*::*cat*	Plasmid (based on pBluescript II SK(+)) harbouring construct for generating knockout of hfq	Cm^R	
p8013_Δ*hfq*::*aphA-1*	Plasmid (based on pBluescript II SK(+)) harbouring construct for generating knockout of hfq	Km^R	This study
p*hfq*-3xFLAG::*aphA-1*	Plasmid (based on pBluescript II SK(+)) harbouring construct for generating a 3xFLAG tagged version of *hfq*	Km^R	This study
p*proq*-3xFLAG::*aphA-1*	Plasmid (based on pBluescript II SK(+)) harbouring construct for generating a 3xFLAG tagged version of ProQ	Km^R	This study
pETM14_H6-3C-NmPNPase	H6-3C-PNPase expression plasmid	KmR	This study

Supplementary Table 4: DNA Oligonucleotides used in this study. Sequences are given in the 5’ → 3’ direction.

Name	Sequence	Purpose
**For detection of sRNAs and mRNAs by northern blot analysis**		
JVO-13786	TCGCTGGTTTGTACGTCCTGAAAA	NMnc006
JVO-13282	AAACTATTAACTGACTACTCGAAC	RocF1
JVO-13780	CGGGTTCAGGGAAACGCTTTCAAT	NMnc034
JVO-13289	ACGGCACTTGTCTTCCCCCCAAT	NMnc041
JVO-11738	CAGCAAACAAGTAATCTAGATTTC	AniS
JVO-14113	TTATTCAGCCTTTATAATACTTGGAC	Bns1
JVO-13920	TCATCGGCGCTGAATCGTTTCACG	5SrRNA
1313	GGAAAGTTTTAACGTGTTTG	*pnp*
1471	GCTAATAAGGTTTTCATATT	*sodC*
1391	TTCAGTTTGGTTTCTTTGTAC	*rpmG*
For T7 transcription of RNAs for electromobility shift assays and PNPase purification		
1418	gttttttTAATACGACTCACTATAGGGAGGATGAAATCTAGATTACTTGTTTG	PCR template for *in vitro* transcription of aniS; carries T7 promotor
1419	AAAAAAAAGGGGTGGCGG	PCR template for *in vitro* transcription of aniS
1420	gttttttTAATACGACTCACTATAGGGAGGACAAGTCCAAGTATTATAAAG	PCR template for *in vitro* transcription of Bns1, carries T7 promotor
1421	AAAAAAGCAGATATATTCGG	PCR template for *in vitro* transcription of Bns1
1422	gttttttTAATACGACTCACTATAGGGAGGATGCAAGAGCTTTTTCAGGA	PCR template for *in vitro* transcription of NMnc0006, carries T7 promotor
1423	AAAAAGACAAAAGCACCCAATAA	PCR template for *in vitro* transcription of NMnc0006
1424	gttttttTAATACGACTCACTATAGGGAGGCAACCCAAACCATTTTTTTGCG	PCR template for *in vitro* transcription of

		NMnc0034, carries T7 promotor
1425	AAATCCAATCAAAAAAGCGTGA	PCR template for *in vitro* transcription of NMnc0034
1426	gtttttttTAATACGACTCACTATAGGGAGGGGTCATATCCGCGCCGCGTA	PCR template for *in vitro* transcription of NMnc0041,carries T7 promotor
1427	AACAAAAGAATGCCGTCCGAACG	PCR template for *in vitro* transcription of NMnc0041
1428	gtttttttTAATACGACTCACTATAGGGAGGTGGAATTGCTTTCAGCGTCCG	PCR template for *in vitro* transcription of pnp, carries T7 promotor
JVO-1445	AAAAAAATGCACACATTCAATAGAAT	PCR template for *in vitro* transcription of RocF1
JVO-1444	gtttttttTAATACGACTCACTATAGGGAGGGTTAGCTGGTTCGAGTAGTC	PCR template for *in vitro* transcription of RocF1, carries T7 promotor
1429	GGTGTGGTTGCCGTATTG	PCR template for *in vitro* transcription of pnp
1463	gtttttttTAATACGACTCACTATAGGGAGG GTTGCCCGCAAACACGTAGT	PCR template for *in vitro* transcription of *rpmG*, carries T7 promotor
1480	GATAAAAATAAAAAAGCCTCCGAAC	PCR template for *in vitro* transcription of *rpmG*
1478	gtttttttTAATACGACTCACTATAGGGAGG CTTGGCGGTGGCGGCCCAC	PCR template for *in vitro* transcription of *sodC*, carries T7 promotor
1479	ATATCTAGCAAAAAAGTGCGGTCA	PCR template for *in vitro* transcription of *sodC*

JVO-16889	AAGTTCTGTTCCAGGGGCCCATGTTCGACAAACACGTTAAAACTTTCC	Nm PNPase cloning SLIC in pETM14
JVO-16890	TGTTAGCAGCCGGATCTCAGTTACTCGGCGGCATTTTCCTCAC	Nm PNPase cloning SLIC in pETM14
JVO-16887	CTGAGATCCGGCTGCTAACAAAG	pETM14 amplification primer
JVO-16888	GGGCCCCTGGAACAGAAC	
**For creating and detecting of knockout and complementation strains**		
**For creating 3XFLAG tagged *proQ* (cloned in pBluescript II SK (+) vector)**		
JVO-13234	gttttGGATCCCCGTTGGCACTCGGCATCGAT	Amplification of the *Bam*HI-*Eco*RI upstream fragment to create p*proq*-3xFLAG::*aphA-1*
JVO-13235	gttttGAATTC TTACTATTTATCGTCGTCATCTTTGTAGTCGATATC ATGATCTTTATAATCACCGTCATGGTCTTTGTAGTC TTCTGCTGCGGAAGATTCGGC	
JVO-13236	gttttGAATTCatccccaaacgaaatgccgtc	Amplification of the *Eco*RI-*Hind*III downstream fragment to create p*proQ*-3xFLAG::*aphA-1*
JVO-13237	gttttAAGCTTctgcttattccatcagccgtt	
329	ACCATGATTACGCCAAGC	Verification of inserts in the pBluescript II vector
kb9	AATACGACTCACTATAGGGC	
JVO-11502	GCGTCGGACTGCGCGCCGCCCCCA	Verification of 3XFLAG tagged *proQ* in *N. meningitidis*
JVO-11603	CTGCGAAAGCGAAGACATCCGCGA	
JVO-13238	Cccgcaaggcttcctgtccaa	Sequencing primer to verify in in-frame fusion of *proQ*::3xFLAG
**For creating and detecting of Δ*proQ and* Δ*hfq* strains (cloned in pBluescript II SK (+) vector)**		
1241	GCGCGCGGATCCTTCGTTGTATTCCTCTAAGAATC	BamHI-HindIII up-stream fragment to create pNHBJ01 for Δ*proQ*
1242	GCGCGCGAAGCTTAAATCGTTGGATTCCGTCGGAGC G	
1243	GCGCGCAAGCTTATCCCCAAACGAAATGCCGTCTGA	HindIII-XhoI downstream fragment to create pNHBJ01 for Δ*proQ*
1244	GCGCGCCTCGAGTTGATCCGCACGGCATCAACGACA	
329	ACCATGATTACGCCAAGC	Verification of inserts in the pBluescript II vector
kb9	AATACGACTCACTATAGGGC	
JVO-13303	GCGCGCAAGCTTTTGATCCGCACGGCATCAACGACA	Verification of 8013 *proQ* deletion in *N.meningitidis*
JVO-13305	CTGCGAAAGCGAAGACATCCGCGA	
1237	GTTTTTTGGATCCCATTTGTCGCCGCCATGAACCGC A	BamHI-EcoRI up-stream fragment to create p8013_Δhfq:: *aphA-1* for Δhfq
1238	GTTTTTGAATTCATTTTTAACTCCGCTATTATGATT	

1239	GTTTTTTGAATTCTCCGCACGAAGCATGACGTGTCAT	EcoRI-HindIII down-stream fragment to create p8013_Δhfq:: *aphA-1* for Δ*hfq*
1240	GTTTTTTAAGCTTCTGTTTGCCGTTTTCGTGTCCCGG	
329	ACCATGATTACGCCAAGC	Verification of inserts in the pBluescript II vector
kb9	AATACGACTCACTATAGGGC	
JVO-11603	CTGCGAAAGCGAAGACATCCGCGA	Verification of 8013 *hfq* deletion in *N. meningitidis*
JVO-11502	GCGTCGGACTGCGCGCCGCCCCCA	
**For construction of Δ*proQ* and Δ*hfq* complementation strains applying overlap PCR**		
JVO-14059	ACCATCCATAGCGGCAGC	Overlap PCR construction ProQ complementation with *erm*C cassette
1395	ACGGCAACATCCGCATTTTGC TCCTTTAGCCTGCCGATGGC	Overlap PCR construction ProQ complementation with *erm*C cassette
JVO-14061	GCAAAATGCGGATGTTGCC	Overlap PCR construction ProQ complementation with *erm*C cassette
JVO-14129	TACCGAGCTCGAATTCCGATTTACTATTTATCGTCGTCATCTTT	Overlap PCR construction ProQ complementation with *erm*C cassette
1396	AGTCTAGTGTGTTAGACTTTAATGTT GCAAAGCCGCAATCCGCTAT	Overlap PCR construction ProQ complementation with *erm*C cassette
JVO-12664	GTCCAAGACTTTCGGCACGGCTTTG	Overlap PCR construction ProQ complementation with *erm*C cassette
JVO-14059	ACCATCCATAGCGGCAGC	Overlap PCR construction Hfq complementation with *erm*C cassette
1394	CATCCACGATTCGATTTTGCTCCTTTAGCCTGCCGATGGC	Overlap PCR construction Hfq complementation with *erm*C cassette
JVO-14651	GCAAAATCGAATCGTGGATG	Overlap PCR construction Hfq complementation with *erm*C cassette
JVO-14129	TACCGAGCTCGAATTCCGATTTACTATTTATCGTCGTCATCTTT	Overlap PCR construction Hfq complementation with *erm*C cassette
1396	AGTCTAGTGTGTTAGACTTTAATGTTTTCGGCGCAAAGCCGCAATC	Overlap PCR construction Hfq complementation with *erm*C cassette
JVO-12664	GTCCAAGACTTTCGGCACGGCTTTG	Overlap PCR construction Hfq complementation with *erm*C cassette
JVO-14059	ACCATCCATAGCGGCAGC	Overlap PCR construction ProQ and Hfq complementation with *erm*C cassette
1265	CATCCACGATTCGATTTTGCTTACTATTTATCGTCGTCATCTTT	Overlap PCR construction ProQ and Hfq complementation with *erm*C cassette
JVO-14651	GCAAAATCGAATCGTGGATG	Overlap PCR construction ProQ and Hfq complementation with *erm*C cassette
JVO-12664	GTCCAAGACTTTCGGCACGGCTTTG	Overlap PCR construction ProQ and Hfq complementation with *erm*C cassette
JVO-14130	ATCGGAATTCGAGCTCGG	Amplification of *ermC* cassette
JVO-14153	AAACATTAAAGTCTAACACACTAG	Amplification of *erm*C cassette
JVO-14067	GTCGCGGCAATGATTTTCTT	Verification of ProQ, Hfq and ProQ/ Hfq complementation
JVO-12664	GTCCAAGACTTTCGGCACGGCTTTG	Verification of ProQ, Hfq and ProQ/ Hfq complementation

**Complementation Strategy for Δ*proQ* and Δ*hfq* applying overlap PCR**					
Mutation	Up-stream region (F1) primers; PCR template	gene (F2) primers; PCR template	Cassette (F3) primers; PCR template	Down-stream region (F4) primers; PCR template	Up-stream-region/ gene/cassette/down-stram-region; PCR template
ProQ +	JVO-14059 1395; 8013 chrom. DNA	JVO-14061 JVO-14129; ProQ-3xFLAG Chrom. DNA	JVO-14130 JVO-14153; Pgcc2	1396 JVO-12664; 8013 chrom. DNA	JVO-14059 JVO-12664; F1, F2, F3, F4
Hfq+	JVO-14059 1394; 8013 chrom. DNA	JVO-14651 JVO-14129; Hfq-3xFLAG Chrom. DNA	JVO-14130 JVO-14153; Pgcc2	1396 JVO-12664; 8013 chrom. DNA	JVO-14059 JVO-12664; F1, F2, F3, F4
ProQ+/Hfq+	JVO-14059 1265; Δ*proQ+proQ* Chrom. DNA			JVO-14651 JVO-12664; Δ*hfq+hfq* Chrom. DNA	JVO-14059 JVO-12664; F1, F2

**Creation of expression plasmids**		
JVO-16887	CTGAGATCCGGCTGCTAACAAAG	pETM14 vector ampl. For SLIC
JVO-16888	GGGCCCCTGGAACAGAAC	pETM14 vector ampl. For SLIC
JVO-16889	AAGTTCTGTTCCAGGGGCCCATGTTCGACAAACACGTTAAAACTTTCC	Nm PNPase cloning SLIC in pETM14
JVO-16890	TGTTAGCAGCCGGATCTCAGTTACTCGGCGGCATTTTCCTCAC	Nm PNPase cloning SLIC in pETM14

Supplementary Table 5: Mapping statistics for *N. meningitidis* strain 8013 UV CLIP-seq. Depicted are the results from two independent experiments (S1, S2) using wild-type (Wt) and a *ProQ::3xFLAG* (FLAG) strain either with (+) or without (-) UV cross-linking.

**Libraries**	**8013_S1 Wt/ +cross-link**	**8013_S1_ FLAG/ - cross-link**	**8013_S1 FLAG/ +cross-link**	**8013_S2 Wt/ +cross-link**	**8013_S2_ FLAG/ -cross-link**	**8013_S2 FLAG/ + cross-link**
No. of input reads	925852	1026412	1430590	793412	527092	880792
No. of reads - PolyA detected and removed	0	0	0	0	0	0
No. of reads - Single 3' A removed	0	0	0	0	0	0
No. of reads – Unmodified	925852	1026412	1430590	793412	527092	880792
No. of reads - Removed as too short	68476	77034	103526	77080	60190	108514
No. of reads - Long enough and used for alignement	857376	949378	1327064	716332	466902	772278
Total no. of aligned reads	547371	610230	912534	418874	256203	491620
Total no. of unaligned reads	310005	339148	414530	297458	210699	280658
Total no. of uniquely aligned reads	174386	224191	380184	152066	103694	251863
Total no. of alignments	1576277	1650583	2475217	1141099	664082	1198255
Total no. of split alignments	0	0	0	0	0	0
Percentage of aligned reads (compared to total input reads)	59.12	59.45	63.79	52.79	48.61	55.82
Percentage of uniquely aligned reads (in relation to all aligned reads)	63.84	64.28	68.76	58.47	54.87	63.66

Supplementary Table 6: Mapping statistics for *N. meningitidis* RNA-seq. Depicted are the results from three independent experiments (#1, #2 and #3) using the wild-type (Wt) and three Δ*proQ* strains.

Libraries	**Δ*proQ* #1**	**Δ*proQ* #2**	**Δ*proQ* #3**	**Wt #1**	**Wt #2**	**Wt #3**
No. of input reads	30190716	27610003	27617526	28026109	28927745	28813057
No. of reads - PolyA detected and removed	0	0	0	0	0	0
No. of reads - Single 3' A removed	0	0	0	0	0	0
No. of reads – Unmodified	30190716	27610003	27617526	28026109	28927745	28813057
No. of reads - Removed as too short	165728	144777	158557	217008	154275	131811
No. of reads - Long enough and used for alignement	30024988	27465226	27458969	27809101	28773470	28681246
Total no. of aligned reads	28374507	25990775	25225102	25629513	26896118	26480879
Total no. of unaligned reads	1650481	1474451	2233867	2179588	1877352	2200367
Total no. of uniquely aligned reads	25872921	23511872	23242486	22940167	24613956	24408279
Total no. of alignments	34698793	32033645	29928414	32029976	32443633	31643346
Total no. of split alignments	0	0	0	0	0	0
Percentage of aligned reads (compared to total input reads)	93.98	94.14	91.34	91.45	92.98	91.91
Percentage of uniquely aligned reads (in relation to all aligned reads)	94.5	94.63	91.86	92.16	93.48	92.33

SUPPLEMENTARY REFERENCES

1 Schoen, C., Kischkies, L., Elias, J. & Ampattu, B. J. Metabolism and virulence in Neisseria meningitidis. *Frontiers in cellular and infection microbiology* **4**, 114, doi:10.3389/fcimb.2014.00114 (2014).

2 Heidrich, N. *et al.* The primary transcriptome of Neisseria meningitidis and its interaction with the RNA chaperone Hfq. *Nucleic acids research* **45**, 6147-6167, doi:10.1093/nar/gkx168 (2017).

3 Dugar, G. *et al.* The CsrA-FliW network controls polar localization of the dual-function flagellin mRNA in Campylobacter jejuni. *Nature communications* **7**, 11667, doi:10.1038/ncomms11667 (2016).

4 Lappann, M., Haagensen, J. A., Claus, H., Vogel, U. & Molin, S. Meningococcal biofilm formation: structure, development and phenotypes in a standardized continuous flow system. *Molecular microbiology* **62**, 1292-1309, doi:10.1111/j.1365-2958.2006.05448.x (2006).

5 Vogel, U. *et al.* Necessity of molecular techniques to distinguish between Neisseria meningitidis strains isolated from patients with meningococcal disease and from their healthy contacts. *Journal of clinical microbiology* **36**, 2465-2470 (1998).

6 Vogel, U., Claus, H., Heinze, G. & Frosch, M. Functional characterization of an isogenic meningococcal alpha-2,3-sialyltransferase mutant: the role of lipooligosaccharide sialylation for serum resistance in serogroup B meningococci. *Medical microbiology and immunology* **186**, 159-166 (1997).

7 Tajima, F. Statistical method for testing the neutral mutation hypothesis by DNA polymorphism. *Genetics* **123**, 585-595 (1989).

8 Kumar, S., Stecher, G., Li, M., Knyaz, C. & Tamura, K. MEGA X: Molecular Evolutionary Genetics Analysis across Computing Platforms. *Molecular biology and evolution* **35**, 1547-1549, doi:10.1093/molbev/msy096 (2018).

9 Zhang, Y. *et al.* Processing-independent CRISPR RNAs limit natural transformation in Neisseria meningitidis. *Molecular cell* **50**, 488-503, doi:10.1016/j.molcel.2013.05.001 (2013).
