# Supplementary material for: The minimal meningococcal ProQ protein has an intrinsic capacity for structure-based global RNA recognition
Source: Nat Commun. 2020 Jun 4;11:2823. doi: 10.1038/s41467-020-16650-6 (PMC7272453; doi:10.1038/s41467-020-16650-6)
Supplement: Supplementary file 4 — Description of Additional Supplementary Files [file 41467_2020_16650_MOESM4_ESM.pdf]

Description of Additional Supplementary Files

File Name: Supplementary Data 1
Description: Full dataset of N. meningitidis ProQ peaks obtained by UV CLIP-seq.

File Name: Supplementary Data 2
Description: Crosslink-specific mutations in N. meninigitidis ProQ peaks obtained by UV CLIP-seq.

File Name: Supplementary Data 3
Description: Comparison of mRNAs directly bound by N. meninigitidis ProQ according to UV-CLIP and N. meninigitidis Hfq as denoted in Heidrich et al. 2017.

File Name: Supplementary Data 4
Description: Full dataset for RNA-seq analysis comparing N. meninigitidis 8013 wild-type (Wt) to proQ deletion strain (ProQ ko) in three independent experiments (exp 1 to 3).

File Name: Supplementary Data 5
Description: COG analysis of RNA-seq data (padj < 0.05, log2 f.c.>1) comparing N. meninigitidis 8013 wt to proQ deletion strain.
